# Claudin-1 Contributes to Gastrointestinal Stromal Tumors (GIST) Resistance to Imatinib Mesylate (IM) via Regulation of FGFR-Signaling

**DOI:** 10.3390/ijms26178138

**Published:** 2025-08-22

**Authors:** Sergei Boichuk, Firyuza Bikinieva, Pavel Dunaev, Aigul Galembikova, Ekaterina Mikheeva, Elena Valeeva, Shinjit Mani, Natalia Khromova, Pavel Kopnin, Leyla Shigapova, Ruslan Deviatiiarov, Elena Shagimardanova, Sergey Ryzhkin, Alexey Sabirov

**Affiliations:** 1 Department of Pathology, Kazan State Medical University, Kazan 420012, Russia; firuza1995@mail.ru (F.B.); dunaevpavel@mail.ru (P.D.); ailuk000@mail.ru (A.G.); miheeva.1973@bk.ru (E.M.); shinjit.mani@gmail.com (S.M.); 2 Department of Radiotherapy and Radiology, Faculty of Surgery, Russian Medical Academy of Continuous Professional Education, Moscow 125993, Russia; rsa777@inbox.ru; 3 Central Research Laboratory, Kazan State Medical University, Kazan 420012, Russia; vevaleeva@yandex.ru; 4 Cytogenetics Laboratory, Carcinogenesis Institute, N.N. Blokhin National Medical Research Center of Oncology, Moscow 115458, Russia; nkhromova@gmail.com (N.K.); pbkopnin@mail.ru (P.K.); 5 Regulatory Genomics Research Center, Institute of Fundamental Medicine and Biology, Kazan Federal University, Kazan 420008, Russia; shi-leyla@yandex.ru (L.S.); ruselusalbus@gmail.com (R.D.); 6 Endocrinology Research Centre, Institute of Personalized Medicine, Moscow 117292, Russia; 7 Graduate School of Medicine, Juntendo University, Tokyo 113-8421, Japan; 8 LIFT—Life Improvement by Future Technologies Institute, Moscow 121205, Russia; rjuka@mail.ru; 9 Genomics and Bioimaging Core Facility, Moscow 121205, Russia; 10 Loginov Moscow Clinical Scientific Center, Moscow 111123, Russia; 11 Tatarstan Cancer Center, Kazan 420029, Russia; a-sabirov@yandex.ru

**Keywords:** gastrointestinal stromal tumors (GIST), imatinib mesylate (IM), claudin 1, resistance

## Abstract

We previously demonstrated that the activation of FGFR signaling in GIST may be a mechanism of GIST resistance to imatinib mesylate (IM). We show here that IM-resistant GIST cells lacking secondary *KIT* mutations overexpress claudin-1 on both transcriptional and translational levels. In contrast, a knockdown of *CLDN1* or inhibition of its activity by PDS-0330 effectively restored GIST’s sensitivity to IM both in vitro and in vivo. This was evidenced by the increased expression of apoptotic markers (e.g., cleaved PARP and caspase-3) and the decreased proliferation rate of IM-resistant GIST T-1R cells treated with a combination of IM and PDS-0330 (or siRNA *CLDN1*). In concordance with these findings, a significant synergy was observed between IM and PDS-0330 in GIST T-1R cells. Importantly, decreased tumor size and weight were observed in IM-resistant GIST xenografts treated with a combination of IM and PDS-0330. Furthermore, the combined treatment of IM-resistant tumors induced an increase in intratumoral apoptosis and other changes, as defined by the histopathologic response rate. Based on the co-immunoprecipitation and immunofluorescence microscopy data, we also demonstrated the strong interaction pattern between CLDN1 and FGFR2. Of note, the inhibition or knockdown of *CLDN1* effectively decreased the phosphorylation of FGFR2 and FRS-2, a well-known FGFR adaptor protein, thereby illustrating CLDN1’s ability to regulate FGFR-signaling and thereby promote FGFR-mediated survival in KIT-inhibited GIST. Consequently, CLDN1 inhibition in GIST effectively disrupted the FGFR-mediated pathway and re-sensitized tumor cells to IM. In concordance with these data, molecular profiling of CLDN1-inhibited GIST T-1R cells illustrated a significant decrease in the majority of FGFR transcripts, including FGFR2, 3, and 4. Additionally, several FGFR ligands (e.g., FGF14, -19, and -23) were also down-regulated in PDS-0330-treated GIST. Notably, exogenous FGF-2 increased CLDN1 expression in a time-dependent manner. In contrast, pan-FGFR inhibitors effectively reduced CLDN1 levels in IM-resistant GIST T-1R cells, thereby illustrating a cross-talk between CLDN1- and FGFR-mediated pathways in IM-resistant GIST. Based on subcellular fractionation and immunofluorescence microscopy data, we also observed partial relocalization of CLDN1 into the cytoplasm in IM-resistant GIST. Notably, PDS-0330 effectively abrogated this relocalization, suggesting that changes in CLDN1 subcellular distribution might also impact GIST resistance to IM. Lastly, based on our small cohort clinical study (n = 24), we observed the increased expression of CLDN1 in most “high-risk” primary GIST known to be associated with poor prognosis and aggressive behavior, thereby illustrating the prognostic value of increased CLDN1 expression in GIST and providing a further rationale to evaluate the effectiveness of CLDN1 inhibition for GIST therapy.

## 1. Introduction

Gastrointestinal stromal tumors (GIST) arise from specialized interstitial cells of Cajal (ICCs) or their precursors, the well-known pacemaker cells that control gut motility. Given that the main driving forces in GIST pathogenesis are the auto-activated, mutant *KIT* receptor tyrosine kinase gene and, less commonly, the platelet-derived growth factor receptor alpha (*PDGFRA*) [1,2,3], targeted therapies are currently accepted as the most effective therapeutic option for adjuvant and non-adjuvant settings, as well. These therapies include imatinib mesylate (IM), a non-selective receptor tyrosine kinase inhibitor (RTKi), which is used as the first-line therapy for GIST patients, with a reported response rate (RR) of ~50–70% and progression-free survival (PFS) of ~20 months [4,5,6]. Following the development of secondary resistance to IM (Gleevec), sunitinib (Sutent), and regorafenib (Stivarga), non-selective kinase inhibitors targeting VEGFR, PDGFR, and c-KIT are recommended for second- and third-line GIST treatment, respectively. Additionally, ripretinib (Qinlock) and avapritinib (Ayvakit) were approved by the FDA in 2020 for late-line therapies of GIST [7,8,9,10,11].

Despite the impressive response rates (mainly observed for first-line targeted therapy), the efficacy of RTKis in GIST eventually declines, indicating the complexity of resistance mechanisms beyond the *KIT/PDGFRA* mutational state. In particular, tumor resistance to RTKi may be due to the activation of alternative RTK-mediated signaling cascades [12,13,14,15,16,17], the increased expression of ABC transporters [18,19,20], the deregulation of apoptosis, and epithelial-to-mesenchymal transition (EMT). The EMT phenotype is involved in regulating the multiple features of cancer cells, including their motility and metastasis formation. EMT also contributes to cancer resistance to targeted therapies, including RTKis. Indeed, the resistance of non-small cell lung cancer (NSCLC) to several RTK inhibitors, including those targeting EGFR-, ALK-, and MET-signaling, has been shown to result from EMT [21,22,23,24,25]. The diverse mechanisms explaining the regulatory role of EMT in NSCLC resistance to TKIs, as mentioned above, are discussed in detail in several reviews [26,27].

Claudins (CLDNs) are a 27-member family of ~25 kDa membrane proteins that govern the barrier properties of tight junctions to form molecular barriers at the paracellular spaces between endothelial and epithelial cells. CLDNs, along with E- and N-cadherins and vimentin, are frequently dysregulated in cancer and are also known as potent regulators of EMT, tumor invasion, and tumor stemness, which are fundamental factors contributing to tumor resistance to chemotherapy. It was postulated that the overexpression of certain CLDNs in cancer inhibits the penetration of chemotherapeutic agents into tumor tissues, leading to chemoresistance. Further studies uncovered the complexity of the molecular mechanisms involved in CLDN-mediated cancer chemoresistance. For example, the regulatory role of CLDN1 in chemoresistance has been proven for several malignancies, including colorectal cancer (CRC) [28] and lung adenocarcinoma [29,30], thereby suggesting that CLDN1 expression in human malignancies may serve as a predictive marker of chemosensitivity. Primeaux V., with co-authors, demonstrated that CLDN1 regulates ephrin type-A receptor 2 (EPHA2), a tyrosine kinase, which activates the AKT signaling pathway and regulates CD44 expression in colorectal cancer (CRC) cells, thereby promoting the stemness of CRC and chemoresistance [31]. CLDN1 was also shown to be a potent factor promoting lung cancer resistance to cisplatin, which was achieved by activating autophagy through the upregulation of Unc-51-like autophagy activating kinase 1 (ULK1) phosphorylation [29]. CLDN3 and 4 were also described as potent regulators of chemoresistance in ovarian and lung cancer, and targeting CLDN3 transcription with small molecules effectively suppressed cancer stemness and reversed their chemoresistance [26,32,33,34]. Moreover, in ovarian cancer cells, CLDN4 was found to interact with both α- and β-tubulins, thereby affecting the structure and dynamic state of the microtubules and reducing apoptotic responses to microtubule-targeting agents (MTAs), including paclitaxel [35]. Opposite effects were observed for ovarian cancer cells, where the knockdown of *CLDN3* and *CLDN4* induced resistance to cisplatin by regulating the Cu transporter CTR1 [36]. CLDN6 was also found to enhance the resistance to doxorubicin in triple-negative breast cancer (TNBC) due to the upregulation of cancer stem cells (CSCs) [37].

Thus, CLDN expression and its role in tumor progression and chemosensitivity were assessed for a broad spectrum of human malignancies. This was analyzed in detail in several fundamental reviews, e.g., [38,39,40]. Despite this, a significant gap remains in the literature regarding the role of CLDNs in GIST pathogenesis, progression, and resistance to TKis. In particular, Hajnalka Gyorffy, with co-authors, reported that mesenchymal tumors also express intercellular junctional proteins. Indeed, various CLDNs were expressed in GIST, angiosarcoma, hemangioma, leiomyosarcoma, and leiomyoma. In particular, CLDN2 was detected in all entities, whereas CLDN1 expression was shown predominantly in leiomyosarcoma. CLDN2, 3, 4, 5, and 7 were expressed on GISTs and leiomyosarcomas [41]. However, the prognostic value of CLDNs expression in GIST pathogenesis and their responsiveness to TKIs remain uncertain.

We demonstrated here that CLDN1 is overexpressed in the IM-resistant GIST T-1R subline, and its knockdown or inhibition by PDS-0330 reversed GIST sensitivity to IM, inducing apoptosis and decreasing proliferation in vitro and in vivo. We also detected a tight physical and functional crosstalk between CLDN1 and FGFR2 in IM-resistant GIST. Indeed, the knockdown of *CLDN1* moderately decreased FGFR2 expression and abrogated FGFR signaling. Conversely, exogenous FGF-2 increased the expression of CLDN1, whereas it declined in the presence of neutralizing anti-FGF-2 mAbs and/or pan-FGFR inhibitors, thereby illustrating the functional partnership between CLDN1 and the FGFR pathway and suggesting a novel regulatory role of CLDN1 in GIST resistance to IM. Notably, this was observed in GIST cells lacking secondary *KIT* mutations and exhibiting activation of the FGFR-mediated pathway. Lastly, CLDN1 expression significantly increased in primary high-risk GIST, which correlated with activation of the FGFR survival pathway, thereby revealing an essential role of CLDN1 in GIST resistance to IM and disease progression.

## 2. Results

### 2.1. An Increased Expression of CLDN1 in GIST T-1 Cells Contributes to IM’s Resistance

**Figure 1:**
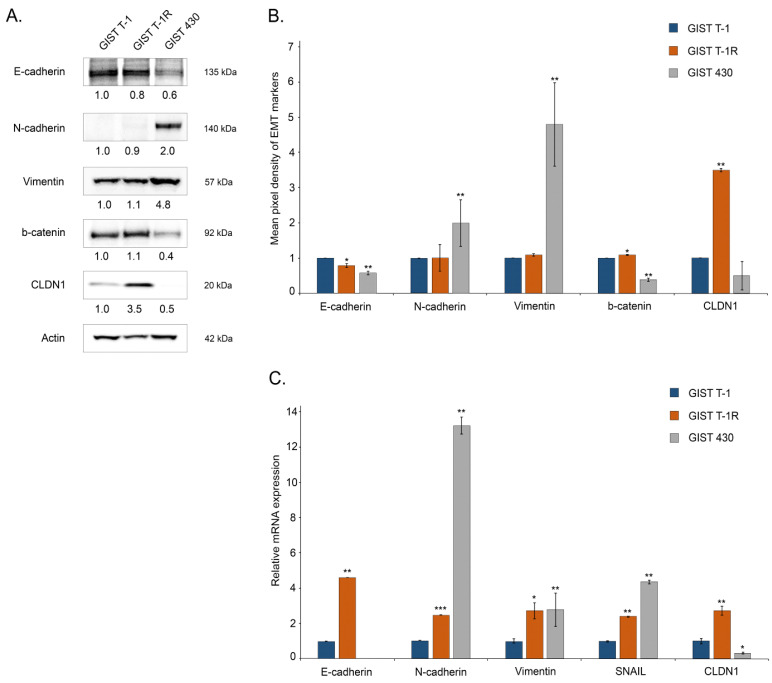
EMT-associated markers in IM-naive (GIST T-1) and IM-resistant (GIST T-1R, GIST 430) cells. (**A**) Expression of EMT-related proteins that were assessed by western blotting; actin staining was used to show the comparable amounts of protein loaded into each sample. (**B**) Quantification by mean pixel density revealed that EMT markers are differently dysregulated in IM-resistant GIST T-1R and GIST430 cells when compared to IM-naïve GIST T-1 cells. (**C**) Changes in the relative expression level of EMT proteins in GIST T-1 vs. T-1R and GIST 430 cells, as determined by quantitative RT-PCR. For internal control, the amplification of actin beta (ACTB) was used. Data are presented as median ± SD. Significant differences with *p* < 0.1 (*), *p* < 0.05 (**), *p* < 0.001 (***) from n ≥ 3 using unpaired Student’s *t*-test.

**Figure 2:**
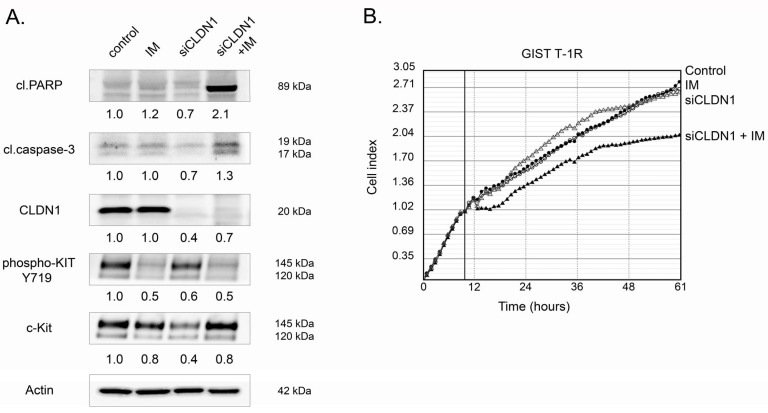
Pro-apoptotic and anti-proliferative activities of siCLDN1 in GIST T-1R cells. (**A**) Cells were treated for 72 h and subjected to western blotting analysis to examine the expression of apoptotic markers, such as cleaved forms of PARP and caspase-3, CLDN1, phospho-KIT Y719, and c-KIT. Actin staining was used to show the comparable amounts of protein loaded into each sample. (**B**) Changes in growth kinetics of GIST-T1R cells, knockdown of *CLDN1*, and treatment with DMSO (control), siCLDN1 alone, and combined with IM. GIST cells (0.5 × 10^5^ /mL) were seeded into the wells of an E-Plate L8 PET cassette and installed in the iCELLigence cell growth kinetics system (ACEA Biosciences, San Diego, CA, USA). Cells were allowed to attach and grow for the following 24 h. Afterwards, IM 1 µM, siRNA CLDN1 alone or in combination, was introduced into the cell culture. DMSO-treated cells served as the control. Cell proliferation index values were recorded every hour throughout the experiment. RTCA Software version 1.0 (ACEA Biosciences, Inc., San Diego, CA, USA) was used to analyze the data.

First, we examined the expression of EMT markers by western blotting (E- and N-cadherins, vimentin, SNAIL) and CLDN1 in IM-naive GIST T-1 and resistant (e.g., GIST T1-R and GIST 430) cells. We found that IM-resistant GIST 430 cells exhibited most of the classical signs of EMT, including the decreased expression of E-cadherin and β-catenin and increased expression of N-cadherin and vimentin. This was observed on transcriptional (Figure 1A,B) and translational (Figure 1C) levels. Additionally, mRNA *SNAIL* was also increased in GIST 430 cells, thereby illustrating that these GIST cells might acquire resistance to IM via diverse molecular mechanisms beyond the well-known secondary *KIT* mutations. Of note, the expression of E-cadherin in GIST T-1R cells did not differ from that of IM-naive GIST cells, whereas PCR data demonstrated this difference. Similarly, vimentin was highly expressed in GIST 430 cells compared to both GIST-T1 and T1-R cells, whereas PCR data showed a moderate increase in this protein. These discrepancies may be due to post-translational modifications of these proteins and differences in protein stability in the various cell lines and sublines. Importantly, in contrast to GIST430 cells, no significant changes in the expression of the aforementioned EMT markers were observed in IM-resistant GIST T-1R cells. However, we found increased CLDN1 expression in this GIST cell subline (Figure 1). This raised the question of whether CLDN1 might be implicated in GIST resistance to IM. To examine this possibility, we knocked down *CLDN1* in GIST T-1R cells with the corresponding siRNA and further treated them with IM. Strikingly, a significant increase of cleaved PARP, a well-known marker of apoptosis, was found (Figure 2A). Similarly, the expression of cleaved caspase-3 significantly increased in IM-treated GIST T-1R cells that had been previously transfected with siRNA against *CLDN1*, thereby illustrating the regulatory role of CLDN1 in its sensitivity to IM. Of note, KIT phosphorylation significantly decreased in IM-treated GIST T-1R cells. Despite this fact, the expression of cleaved PARP and caspase-3 was not detected, thereby revealing alternative molecular pathways that regulate their survival, which underlie mechanisms beyond KIT signaling, as shown previously [12]. In concordance with these findings, the knockdown of *CLDN1* in GIST T-1R cells significantly decreased their proliferation capacities when used in combination with IM. In contrast, IM treatment alone or the knockdown of *CLDN1* without KIT inhibition did not slow down the proliferation rate of these cells (Figure 2B). In agreement with these data, crystal violet staining of GIST T-1R cultures treated with IM in the presence of *siRNA CLDN1* also revealed a substantial decrease in cellular viability, whereas treatment with IM alone did not have a significant impact on GIST’s viability and proliferative activity (Supplementary Figure S1A,B). In concordance with these findings, IC50 values for IM were decreased in GIST T-1R cells after the knockdown of *CLDN1* (Supplementary Figure S2).

**Figure 3:**
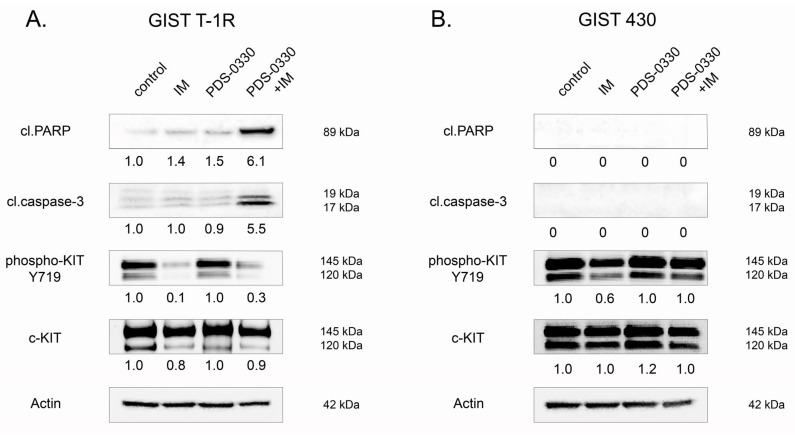
Inhibition of CLDN1 with PDS-0330 in GIST T-1R and GIST 430 cells. GIST T-1R (**A**) and GIST 430 (**B**) cells were treated with PDS-0330 (5 µM) or imatinib (IM) (1 µM) alone or in combination for 72 h and subjected to western blotting analysis to examine the expression of apoptotic markers, such as cleaved forms of PARP and caspase-3, phospho-KIT Y719, and c-KIT. Actin staining was used to show the comparable amounts of protein loaded into each sample.

**Figure 4:**
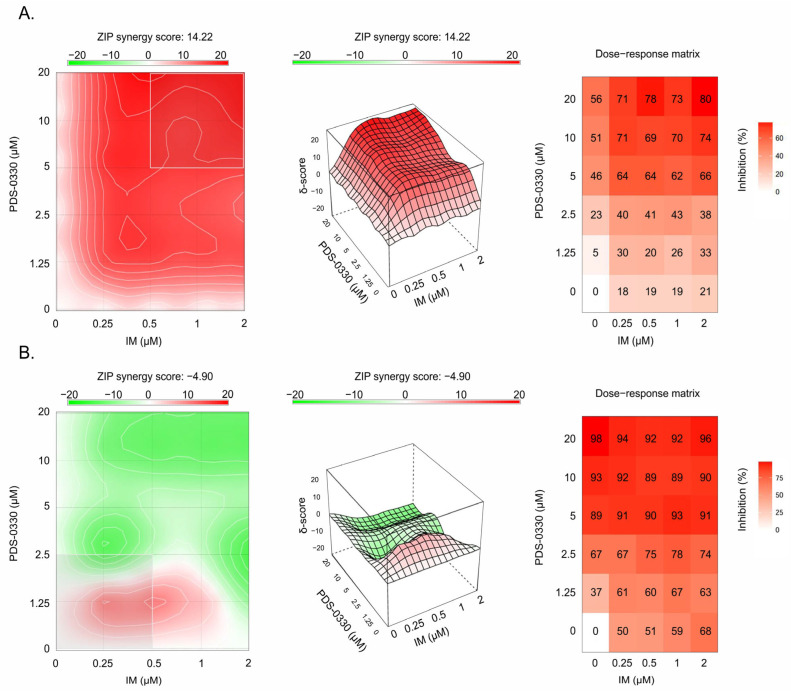
Assessment of the synergy between IM and PDS-0330 observed for IM-resistant GIST T-1R (**A**) and GIST 430 (**B**) cells. Potential additive, antagonistic, and synergistic effects of PDS-0330 on IM were calculated based on cell viability under the influence of PDS-0330, IM, and their combination using the R package of the computational tool Synergy Finder. Results were obtained by calculating the synergy scores via a ZIP computational tool. A value of synergy score (SC) of PDS-0330 and IM combinations < −10 was considered an antagonistic effect, whereas SCs between −10 and 10 and SCs > 10 were considered an additive and synergistic effect, respectively.

Similarly, we observed that PDS-0330, a specific CLDN1 inhibitor, effectively sensitized GIST T-1R cells to IM. This was evidenced by increased apoptosis in GIST T-1R cells treated with IM in combination with PDS-0330 (Figure 3A). As expected, when PDS-0330 and IM were used alone, the expression of the aforementioned apoptotic markers was not detected (Figure 3A). Importantly, PDS-0330 failed to sensitize GIST 430 cells to IM (Figure 3B), thereby revealing an essential role of overexpressed CLDN1 in GIST resistance to IM. In concordance with these findings, a prominent synergy between IM and PDS-0330 was observed in GIST T-1R cells. This was evidenced by calculating the synergy scores (SCs) by using the R package of the computational tool Synergy Finder (Software 3.16.0) (Figure 4A). In contrast to GIST T-1R cells, GIST 430 cells were not sensitive to the combination of CLDN1- and KIT-inhibitors (Figure 4B). Similar results were obtained by calculating the synergy scores via four different computational tools (i.e., the zero interaction potency (ZIP), the highest single agent (HSA), Bliss, or Loewe). Indeed, the data shown in Table 1 illustrate a significant synergy (e.g., SC > 10) between IM and PDS-0330 in IM-resistant GIST T-1R cells, whereas no synergy was found between IM and PDS-0330 in IM-resistant GIST 430 cells. Indeed, SCs for IM and PDS-0330 in GIST430 cells were even negative when calculated by ZIP, Bliss, or Loewe tools, whereas the HSA program resulted in SC values < 10 (Table 1). Thus, we concluded that high SC values between IM and PDS-0330 found in GIST T-1R might be due to the increased expression of CLDN1 (Figure 1). In contrast, the lack of synergy between the IM and PDS-0330 observed in GIST430 cells may be due to the absence of CLDN1 in these particular cancer cells, which acquired resistance to IM via other molecular mechanisms, underlying beyond the CLDN1 pathway and including secondary *KIT* mutations and, potentially, the development of EMT. In agreement with SC data, the proliferation rate of GIST T-1R cells significantly decreased when the cells were treated with a combination of IM and PDS-0330 compared to cells treated with IM or PDS-0330 alone (Supplementary Figure S3A). Again, no inhibitory effect of PDS-0330, used alone or in combination with IM, was observed in GIST 430 cells (Supplementary Figure S3B), suggesting that the increased expression of CLDN1 in IM-resistant GIST is a promising molecular target for personalized therapy in GIST.

Thus, our data indicate that the increased expression of CLDN1 in GIST contributes to IM resistance, and knocking down or inhibiting CLDN1 reverses the IM-resistant phenotype in GIST T-1R cells.

### 2.2. Functional Cross-Talk Between CLDN1 and FGFR-Signaling in GIST

**Figure 5:**
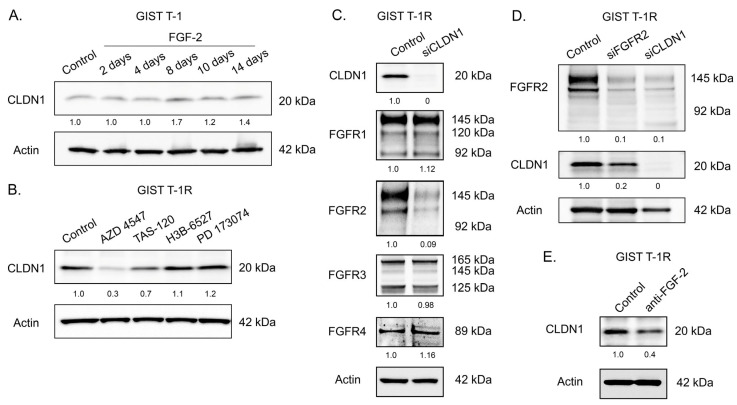
(**A**) Expression of CLDN1 in the GIST T-1 cells treated with FGF-2 (100 ng/mL) for 2 weeks. (**B**) Immunoblot analysis for CLDN1 in GIST T-1R cells treated with solvent (DMSO) (control), AZD 4547 (1 μM), TAS-120 (1 μM), H3B-6527 (1 μM), and PD 173074 (10 μM) for 48 h. Actin stain is used as a loading control. (**C**) Expression of FGFR 1-4 and CLDN1 in GIST T-1R cells transfected with scrambled siRNA (control) and siRNA CLDN1. Actin stain was used as a loading control. (**D**) Expression of FGFR2 and CLDN1 in GIST T-1R cells after the knockdown by corresponding siRNAs. Actin stain was used as a loading control. (**E**) Expression of CLDN1 in control (non-treated) GIST T-1R cells and cells cultured in the presence of neutralizing anti-FGF-2 mAbs (20 μg/mL, 72 h incubation). Actin stain was used as a loading control.

Given that GIST T-1R cells exhibit the signs of activation of the FGFR-signaling pathway, which was previously shown as a potent mechanism of secondary resistance to IM named receptor tyrosine kinase switch [12], and taking into account increased expression of CLDN1 in these particular GIST cells, we examined the potential interplay between these molecular events. For this purpose, we initially used IM-naive GIST T-1 cells, which lack activation of the FGFR pathway [17] and exhibit low basal levels of FGF-2. We found that culturing these cells in the presence of FGF-2 (100 ng/mL) increased CLDN1 expression in a time-dependent manner, which reached its maximal level after 1 week and retained on its high level until the end period of the experiment (14 days), thereby suggesting the potential crosstalk between the activated FGFR-pathway and CLDN1 in GIST (Figure 5A). Despite the moderate nature of this effect, it was reproducible, thereby illustrating the connection between the activation of FGFR signaling and the increased expression of CLDN1 in IM-resistant GIST. Conversely, the inhibition of FGFR-signaling in IM-resistant GIST cells overexpressing FGF-2 effectively decreased CLDN1 expression. This was observed for IM-resistant GIST cells treated with pan-FGFR inhibitors (e.g., AZD 4547 and TAS-120). In contrast, selective inhibition of FGFR1 or FGFR4 signaling pathways by corresponding inhibitors (e.g., PD 170374 and H3B-6527, respectively) failed to decrease CLDN1 levels in IM-resistant GIST (Figure 5B), thereby suggesting the potential crosstalk between CLDN1 and FGFR1 or FGFR2. In agreement with these findings, we also observed that a knockdown of *CLDN1* effectively downregulated the expression of FGFR2. In contrast, the levels of other types of FGF receptors (e.g., FGFR1, FGFR3, and FGFR4) remained unchanged after *CLDN1* knockdown (Figure 5C). Similarly, a knockdown of *FGFR2* moderately decreased the expression of CLDN1 in GIST cells (Figure 5D), thereby illustrating a tight connection between the proteins mentioned above. Lastly, we found that the interruption of FGF-signaling with neutralizing anti-FGF-2 mAbs in GIST T-1R cells also induced a moderate decrease in CLDN1 expression (Figure 5E), thereby revealing a close functional cross-talk between the FGFR pathway and CLDN1 in this particular IM-resistant GIST cell line.

### 2.3. CLDN1 Interacts with FGFR2 and Regulates FGFR-Signaling in IM-Resistant GIST

**Figure 6:**
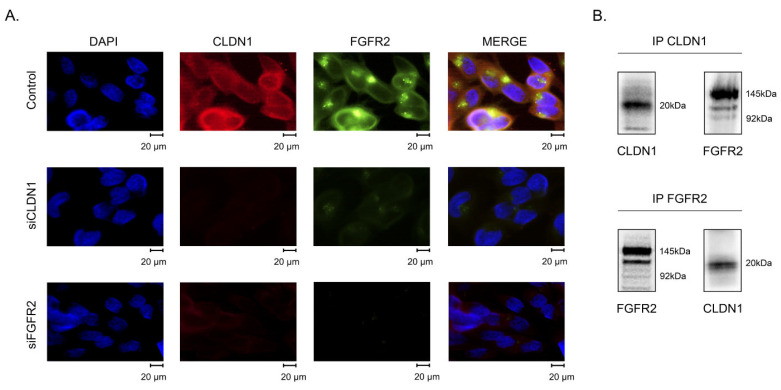
(**A**) Knockdown of *CLDN1* and *FGFR2* genes was performed in GIST T1-R cells using the corresponding small interfering RNAs (siCLDN1, siFGFR2), and cells were subjected to immunofluorescence staining for FGFR2 (green) and CLDN1 (red) at 72 h post-transfection. To outline the nucleus, the images were also merged with DAPI staining (blue): magnification 100×, scale bars 20 µm. The images were examined in at least five fields of view (70–80 cells were captured for each particular experimental setting). (**B**) Co-immunoprecipitation (co-IP) assays were used to demonstrate the interaction between FGFR2 and CLDN1 in IM-resistant GIST T1-R cells. The lysates were immunoprecipitated with anti-CLDN1 (up) or anti-FGFR2 mAbs (bottom) and probed for their interaction partners to illustrate the formation of the endogenous FGFR2-CLDN1 complex.

To examine whether FGFR2 interacts with CLDN1 in IM-resistant GIST, we initially performed immunofluorescence staining for FGFR2 and CLDN1 and found high levels of their co-localization (Figure 6A). Notably, when the corresponding siRNA knocked down *CLDN1*, a significant decrease in FGFR2 was observed (Figure 6A, medium panel). Similarly, a knockdown of *FGFR2* decreased CLDN1 expression in IM-resistant GIST (Figure 6A, bottom panel), thereby suggesting a tight connection between these proteins. This was in agreement with the co-immunoprecipitation (co-IP) assay, which illustrated the direct interaction between FGFR2 and CLDN1 in GIST lysates precipitated with anti-CLDN1 mAbs and probed for FGFR2 (Figure 6B). Importantly, we observed a similar interaction pattern between FGFR2 and CLDN1 during the reverse co-IP assay, when cellular lysates were precipitated with anti-FGFR2 monoclonal antibodies (mAbs) and probed for CLDN1.

Thus, we showed here the tight co-localization and interaction patterns between FGFR2 and CLDN1 in IM-resistant GIST, which acquired its resistance to this RTKi due to activation of the FGFR-signaling pathway [12]. In this case, CLDN1 may be a crucial factor in maintaining effective FGFR signaling and regulating survival in KIT-inhibited GIST cells. Thus, we proposed that a knockdown of *CLDN1* or its inhibition by PDS-0330 disrupts the FGFR-mediated survival cascade in IM-resistant GIST, thereby providing a possible explanation for their re-sensitization to IM, as shown above (e.g., Figure 2, Figure 3 and Figure 4).

To examine this possibility directly, we assessed the activation of the FGFR-signaling pathway in *CLDN1* knockdown or inhibited GIST T-1R cells. We found that PDS-0330 significantly decreased the expression of phosphorylated (i.e., activated) FGFR1/2 in IM-resistant GIST (Supplementary Figure S4). Similarly, the expression of phosphorylated fibroblast growth factor receptor substrate 2 (FRS-2) was decreased after CLDN1 inhibition. Of note, similar results were obtained after *CLDN1* knockdown, thereby revealing CLDN1’s ability to maintain effective FGFR-signaling in IM-resistant GIST and thereby promoting FGFR-mediated survival in KIT-inhibited GIST.

### 2.4. CLDN1 Regulates the Motility of IM-Resistant GIST

**Figure 7:**
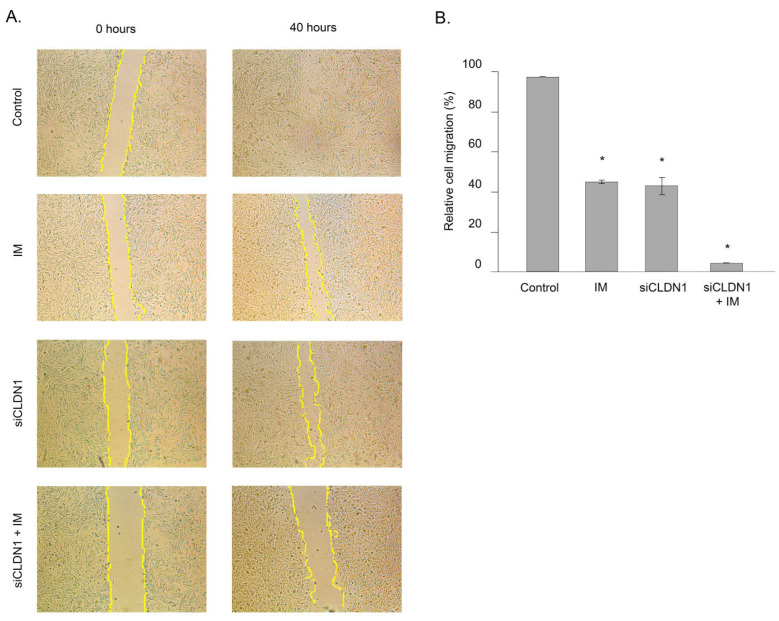
*CLDN1* knockdown potentiates IM-induced decrease in migration of IM-resistant GIST T-1R cells. (**A**) Representative images of the wound healing assay of GIST T-1R cells upon IM treatment (1 µmol/L) for 40 h alone or previously transfected with siRNA *CLDN1*. GIST cells treated with a vehicle were used as controls. Scale bar = 500 μm (**B**) Quantitative analysis of wound area GIST T-1R cells treated with DMSO (control), IM, or *siRNA CLDN1* alone or in combination. Data are presented as median ± SD. Significant differences with *p* < 0.05 (*) from n ≥ 3 using unpaired Student’s *t*-test.

To examine whether CDLN1 has an impact on IM-resistant GIST’s motility, we knocked down *CDLN1* in IM-resistant GIST T-1R cells with corresponding siRNA and performed a scratch-wound healing assay to study tumor cell migration ability. We observed that the migration of GIST cells at the edge of the scratch was slightly decreased following IM treatment when compared to the non-treated control (Figure 7A). As expected, the knockdown of *CLDN1* provided a moderate inhibitory effect on the migration of IM-resistant GIST. Moreover, IM treatment of GIST cells that had been previously transfected with siRNA against CLDN1 dramatically reduced their migration (Figure 7B). Notably, we cannot rule out the possibility that this inhibitory effect may also be attributed to the decreased viability of IM-resistant GIST cells that were previously knocked down for *CLDN1* and subsequently treated with IM.

### 2.5. Subcellular Distribution of CLDN1 in IM-Resistant GIST

**Figure 8:**
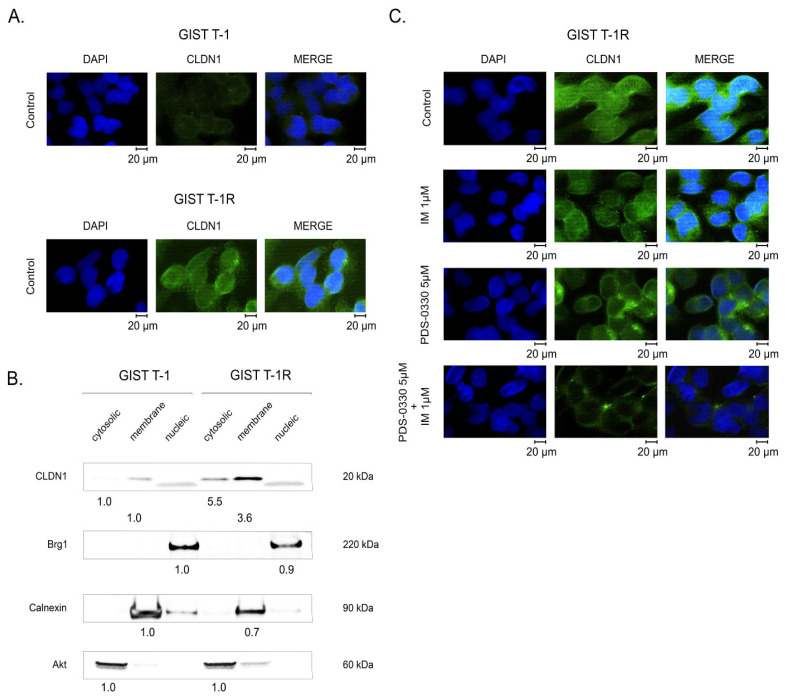
Subcellular distribution of CLDN1 in IM-naive (GIST T-1) and -resistant (GIST T-1R) cells. (**A**) GIST cells were stained for CLDN1 (green), as described in the Materials and Methods section. DAPI stain (blue) was used to outline the nuclei. The representative images were captured by the fluorescence Olympus BX63 microscope and Spot Advanced Imaging System. Magnification 100×, scale bars 20 μm. The images were examined in at least five fields of view (70–80 cells were captured for each particular experimental setting). (**B**) Cytoplasmic, membrane, and soluble nuclear protein fractions of GIST cells were purified using the Subcellular Protein Fractionation Kit, as described in the Materials and Methods section, and subjected to immunoblotting. The membranes were probed for CLDN1. The purity of the fractions, as mentioned above, was verified by probing for Brg1 (nuclear), calnexin (membranous), and AKT (cytoplasmic) markers. (**C**) PDS-0330 disrupts the translocation of CLDN1 from the membrane to the cytoplasm in KIT-inhibited GIST. The cells were treated with IM, PDS-0330 alone, or in combination for 72 h and further processed for immunofluorescence staining with an anti-CLDN1 antibody (green). DAPI nuclear staining (blue) was used to outline the nuclei. Images were captured using an Olympus BX63 fluorescence microscope. Magnification 100×, scale bars 20 μm. The images were examined in at least 5 fields of view (70–80 cells were captured for each particular experimental setting).

Given that changes in the subcellular distribution of CLDN1 are shown to be an independent marker of aggressiveness and poor outcome in a broad spectrum of human malignancies (e.g., colon and triple-negative breast cancer, urothelial carcinoma), we initially compared the subcellular distribution of CLDN1 between IM-sensitive and -resistant GIST. In agreement with our previous findings, we found that CLDN1 was exclusively expressed on the membranes of IM-naive GIST T-1 cells, whereas in IM-resistant GIST T1-R cells, it was translocated to the cytoplasm (Figure 8A). Subcellular fractionation data revealed the appearance of CLDN1 in the cytoplasmic compartment of IM-resistant GIST cells (Figure 8B). We also observed the abundant expression of CLDN1 in the membranous fraction in IM-resistant cells when compared to IM-naive GIST (Figure 8B).

Interestingly, PDS-0330 effectively abrogated CLDN1’s shuttling from the membrane compartment to the cytoplasm. Of note, this was observed when the CLDN1 inhibitor was combined with IM, whereas no changes in the subcellular distribution of CLDN1 were detected in GIST cells treated with PDS-0330 alone (Figure 8C). As expected, when IM was used alone, no changes in the subcellular distribution of CLDN1 were observed.

Thus, we found elevated levels of CLDN1 in both the cytoplasmic and membranous compartments in GIST T-1R cells compared to GIST T-1 cells. This is in concordance with the increased expression of total CLDN1 in GIST T-1R cells, as shown in Figure 1A. The fractionation data were in concordance with the immunofluorescence staining data, shown in Figure 8A, revealing that, besides the increased expression of CLDN1 in the membranes, this protein is also significantly increased in the cytoplasmic compartment.

Overall, our data illustrate the significant changes in CLDN1 subcellular distribution in IM-resistant GIST and the ability of PDS-0330 to prevent its trafficking from the membranes into the cytoplasmic compartments. The last one might also be relevant to the resensitization of IM-resistant GIST to IM in cases of CLDN1-mediated signaling inhibition. Further studies are needed to examine this possibility directly.

### 2.6. Targeting of CLDN1 In Vivo Inhibits the Growth of KIT-Inhibited IM-Resistant Xenografts

**Figure 9:**
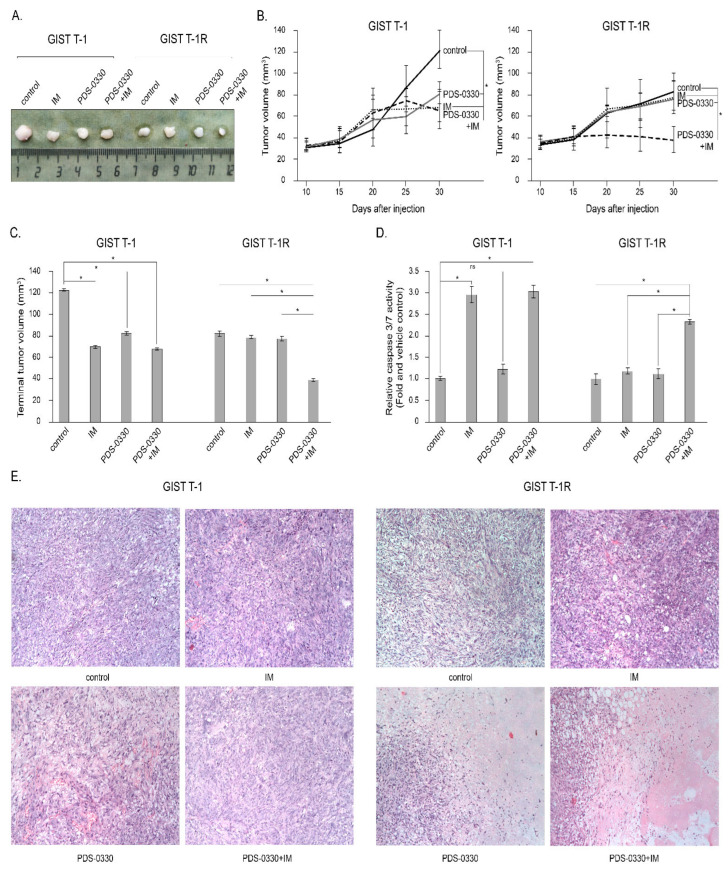
Anti-tumor effects of CLDN1 inhibitor PDS-0330 and IM in nude mice xenograft GIST models. After the subcutaneous inoculation of IM-naive (GIST T-1) and IM-resistant (GIST-T-1R) cells (day 14), nude mice were randomized into four groups (n = 4). They administered i.p. 100 μL of vehicle (negative control), PDS-0330 (5 mg/kg), IM (50 mg/kg), or a combination of PDS-0330 and IM, three times a week, for 2 weeks. The changes in tumor sizes were calculated as a percentage of the baseline. The tumor volume in each group was assessed using calipers every 5 days for a total of 30 days, calculated as length × width × height × 0.5, and tumor growth curves were drawn accordingly. Results were expressed as the mean tumor volume compared to the control (vehicle-treated) animals. (**A**) Representative tumors are shown for each experimental group. (**B**) Changes in the tumor volume of IM-naive (GIST T-1) and -resistant (GIST T-1R) xenografts. (**C**) The tumor volumes at the end-point of the experiment (2 weeks of post-treatment) for each experimental group. (**D**) Relative activity of Caspases-3/7 in GIST xenografts treated as indicated above. Data are presented as median ± SD. Significant differences with *p* < 0.05 (*), ns—non-significant from n = 4 using unpaired Student’s *t*-test. (**E**) Representative images of hematoxylin and eosin-stained IM-naïve and -resistant GIST xenografts treated for 14 days. Scale bar = 500 μm.

To corroborate the in vitro studies, we examined whether CLDN1 inhibitors used alone or in combination with IM exhibited anti-tumor activity against IM-naive and -resistant GIST xenografts. For this purpose, GIST T-1 vs. T1-R cells were injected into the flanks of female adult athymic nude mice, and tumors were allowed to grow for at least 2 weeks before single (e.g., PDS-0330 or IM) or dual treatment. Representative tumors of each experimental group are shown in Figure 9A. As expected, IM inhibited the growth of IM-naïve GIST xenografts, whereas a minor inhibitory effect was observed for IM-resistant GIST after IM treatment (Figure 9B,C). Although PDS-0330 exhibited minor anti-tumor activities against both GISTs, its combination with IM significantly inhibited the growth of IM-resistant GIST xenografts over 2 weeks of treatment when compared to the baseline (Figure 9B,C). We also observed an increase in caspase-3 and -7 activities in IM-resistant GIST xenografts treated with a combination of IM and PDS-0330 (Figure 9D). As expected, the significant activity of caspases 3 and 7 was observed in IM-naïve GIST xenografts treated with IM alone or in combination with PDS-0330 on day 6 post-treatment. In contrast, the CLDN1 inhibitor used alone was not effective in both IM-naïve and resistant xenografts (Figure 9D). Lastly, the histological examination of tumor xenografts revealed the significant enlargement of necrotic areas in IM-resistant GIST treated with IM in combination with PDS-0330 for 14 days, compared to non-treated controls or single-treated animals (Figure 9C).

Thus, our data revealed that the inhibition of CLDN1 signaling in IM-resistant GIST re-sensitizes them to IM in vivo, thereby illustrating a novel mechanism contributing to GIST resistance to this first-line targeted drug.

### 2.7. Molecular Profiling of IM-Resistant GIST Cells with Inhibited CLDN1 Signaling

**Figure 10:**
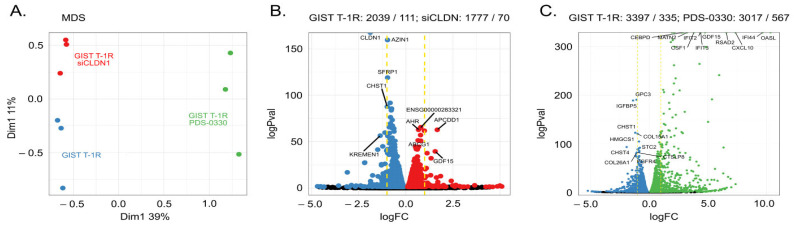
Transcriptional alterations in GIST cell lines. (**A**) MDS (multidimensional scaling) plot on TMM (trimmed means of M values) normalized counts. All three samples are segregated by the first two dimensions. (**B**) Differential expression (DE) results between GIST T-1R cells and the knock-down cell line. The top DE genes are labeled. Dashed lines indicate thresholds for differentially expressed (DE) gene selection FRD < 0.05 and |logFC| > 2. Red and blue colored dots correspond to DE genes up-regulated in GIST T-1R cells and the knock-down cell line, respectively. (**C**) Differential expression (DE) results between GIST T-1R cells and GIST T-1R cells treated with PDS-0330. The top DE genes are labeled. Dashed lines indicate thresholds for differentially expressed (DE) gene selection FRD < 0.05 and |logFC| > 2. Blue and green colored dots correspond to DE genes up-regulated in GIST T-1R cells and GIST T-1R cells treated with PDS-0330, respectively.

To investigate the potential molecular mechanisms underlying CLDN1-mediated resistance to IM in GIST, we conducted a comparative analysis of the whole transcriptome in mock-transfected, siCLDN1-transfected, and PDS-0330-treated GIST cells. Indeed, profound differences in the transcriptome profiling were observed for siCLDN1-transfected GIST cells when compared to mock-transfected (i.e., control) cells (Figure 10A,B). As expected, *CLDN1* was increased in GIST T-1R cells (Figure 10B). Additionally, *AZIN1* (antizyme inhibitor 1), *SFRP1* (soluble frizzled related protein 1), and *CHST1* (carbohydrate sulfotransferase 1) were up-regulated in mock-transfected GIST cells when compared to *CLDN1*-knockdown GIST (Figure 10B). In particular, *SFRP1* is known as a potent modulator of Wnt signaling due to its ability to interact directly with Wnt. SFRP1 exhibits multiple effects that may be involved in regulating the chemosensitivity of cancer cells. This includes the anti-proliferative effects of SFRP1, which delays the G1 phase and entry into the S phase, as well as inhibiting tubule formation. Similarly, significant differences between DMSO- and PDS-0330-treated GIST cells were found for CSH1 and CSH4 transcripts (Figure 10C). Moreover, *GPC3*, *HMGCS1*, *COL26A1*, *COL15A1*, *STC2* transcripts were found to be decreased in CLDN1-inhibited cells. This was also associated with increased levels of *IFIT2*, *IFIT5*, *CEBPD*, *MATN2*, *GDF15*, and *OASL* transcripts when compared to DMSO-treated cells.

**Figure 11:**
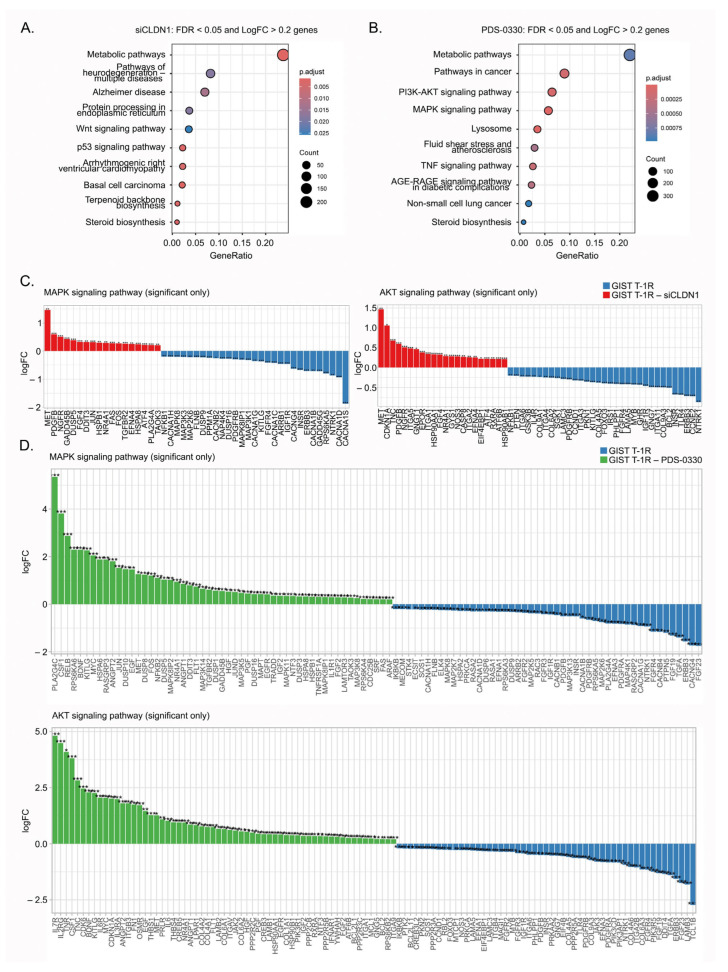
(**A**,**B**) Pathway enrichment analysis for DE genes; top 10 pathways are shown. *p*-values were corrected with the Benjamini–Hochberg procedure. (**C**) Gene expression changes in PI3K/Akt, MAPK signaling pathways in GIST T-1R cells and the knock-down cell line. *** FDR < 0.001, ** FDR 0.001–0.01, * FDR 0.01–0.05, not significant FDR > 0.05. (**D**) Gene expression changes in PI3K/Akt, MAPK signaling pathways in GIST T-1R cells and GIST T-1R cells treated with PDS-0330. *** FDR < 0.001, ** FDR 0.001–0.01, * FDR 0.01–0.05, not significant FDR > 0.05.

Based on the literature data (as discussed below), some of these hits may be involved in regulating tumor behavior, including their sensitivity to chemotherapy. In particular, this belongs to the metabolic, WNT-, and p53-mediated signaling pathways, which were shown to be altered after knockdown of *CLDN1* (Figure 11A). Notably, the significant differences between mock-treated and PDS-0330-treated GIST cells were also associated with metabolism, cancer, MAPK, AKT, and TNF signaling (Figure 11B). Of note, the dysregulation of genes involved in the metabolic pathways was at the top in both CLDN1-inhibited GIST and PDS-0330-treated GIST, as well. This is in concordance with recent findings illustrating the regulatory role of CLDN1 in the metabolism of pancreatic cancer cells, which in turn has a significant impact on their sensitivity to certain chemotherapies [42].

Given that GIST T-1R cells exhibited activation of the FGFR-signaling pathway [12], we examined the impact of CLDN1 down-regulation and/or its inhibition on the FGF/FGFR transcripts. We found that down-regulation of FGFR4 and FGFR1OP2 was the most prominent change in siCLDN1-transfected GIST cells, as well as overexpression of FGF4 (Supplementary Figure S5A). Similarly, PDS-0330 GIST cells exhibited a significant decrease in the majority of FGFRs, including FGFR2, 3, and 4. Additionally, FGF14, 19, and 23 ligands were also down-regulated in PDS-0330-treated GIST cells (Supplementary Figure S5B).

The impact of CLDN1 inhibition or knockdown on the activity of downstream signaling pathways involved in maintaining GIST survival and proliferation was also examined. For this purpose, we also looked over the changes in MAPK- and AKT-signaling pathways (Figure 11C,D). A detailed analysis of the changes in the transcriptome profile of CLDN1-inhibited GIST and its potential impact on the behavior of cancer cells, including their sensitivity to chemotherapy, is described in more detail in the Discussion section.

### 2.8. Clinical Significance of CLDN1 in Primary GIST

**Figure 12:**
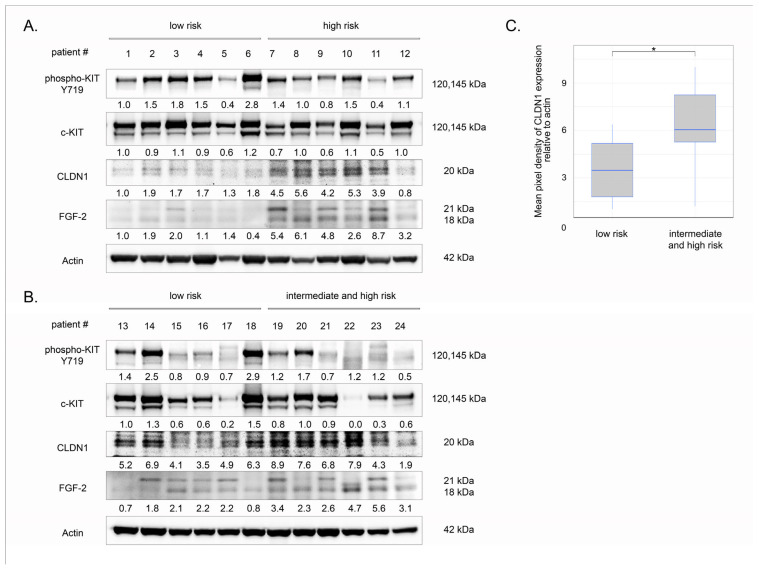
(**A**,**B**) Changes in CLDN1 expression in primary GIST with different prognostic groups (n = 24). Western blotting of lysates from GIST patients was used to examine the expression of CLDN1, FGF-2, and phosphorylated and total forms of KIT. Actin staining was used to show the comparable amounts of protein loaded into each sample. (**C**) Changes in CLDN1 expression in primary GIST with different prognostic groups (n = 24). Descriptive and analytical statistical data of two comparison groups (low risk/intermediate and high risk). Significant differences with *p* < 0.05 (*) using unpaired Student’s *t*-test. Box plots are presented as median (quant 25; quant 75). Low risk 3.45 (1.79; 5.17); intermediate and high risk 6.02 (5.26; 8.24).

To determine the prognostic value of CLDN1 expression in GIST, we examined primary tumors by western blotting. Seventy-five percent of patients were female. The median age of patients was 62 years (62.0 ± 12.2). The most frequent location of GIST was the stomach (83%), followed by the small bowel (13%), and the lesser omentum (4%). Ninety-two percent of tumors were positive for KIT IHC staining. The detailed characteristics of GIST patients are shown in Supplementary Table S1. The IHC-staining data correlated with Western blot results, shown in Figure 12A,B. Indeed, among the 24 GIST patients enrolled in this study, two patients were found to be negative for c-KIT expression (patients #17 and 22). According to the well-known relapse risk criteria (e.g., Miettinen’s criteria) based on the AFIP classification [43,44], 50% of samples were considered to be in the low risk group, 8% belonged to the intermediate risk group, and 42% of GISTs were regarded as high risk.

Despite the absence of correlation between CLDN1 expression and tumor location (stomach/other localization) (*p* = 0.34161; two-tailed Fisher’s exact test), gender (female/male) (*p* = 1.00000; two-tailed Fisher’s exact test), and age (*p* = 0.59547; Student’s *t*-test), a significant correlation between CLDN1 expression in GIST specimens and the prognostic group (low risk/intermediate and high risk) was observed (*p* = 0.02123; two-tailed Fisher’s exact test).

**Figure 13:**
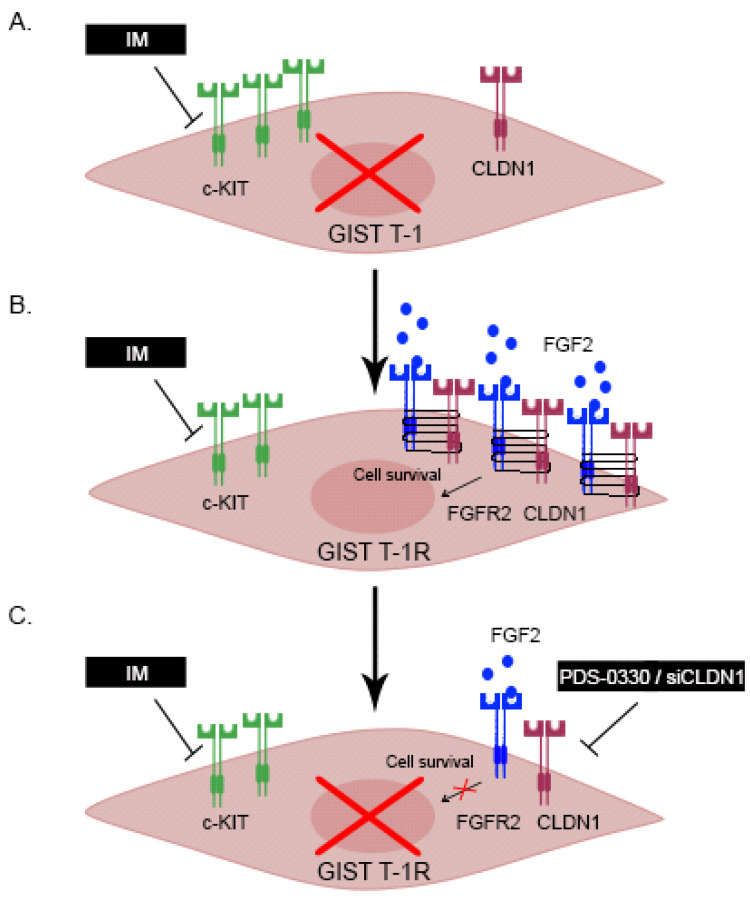
Inhibition of CLDN1-signaling in IM-resistant GIST increases their sensitivity to IM. (**A**) IM induces cell death of IM-naïve GIST T-1 cells exhibiting high levels of phospho-KIT, whereas expression of CLDN1 is low; (**B**) IM-resistant GIST T-1R cells acquire resistance to IM via an autocrine mechanism due to FGF-2-induced activation of FGFR-signaling. CLDN1 expression increases, whereas phospho-KIT expression moderately decreases. (**C**) Inhibition or knockdown of *CLDN1* decreases the expression of FGFR2 and sensitizes IM-resistant GIST T-1R to IM.

Indeed, we found that CLDN1 expression increased in most of the tumor specimens from intermediate- to high-risk GIST. Indeed, 10 tumor specimens were found to be strongly positive for CLDN1 expression among 12 high-risk GIST samples being analyzed. This is illustrated in the representative Western blot images shown in Figure 12A,B. In contrast, the majority (e.g., 9 of 12 samples) of low-risk GIST specimens exhibited minor expression of CLDN1. This was also summarized in Figure 13C to illustrate that > 75% of low-risk GIST specimens (n = 12) showed decreased expression of CLDN1 (densitometry analysis of CLDN1 expression was below < 5.2 after the normalization of GIST samples to the expression of actin).

To illustrate the significant differences in CLDN1 expression between high- and low-risk GIST, the original images of CLDN1 and actin were processed in the Image J program and statistically analyzed for each GIST sample, as shown in Table 2. Overall, these data demonstrate the clinical and prognostic significance of increased CLDN1 expression in primary GIST.

Lastly, we examined the potential connection between CLDN1 and FGFR2 and its ligand in high- and low-risk primary GIST. Although we did not observe significant changes in FGFR2 expression between low- and high-risk primary tumors, we found a substantial increase in FGF-2 in high-risk tumors that exhibited high levels of CLDN1. In contrast, low-risk malignancies lacking CLDN1 expression exhibited only basal levels of FGF-2 (Figure 13), indicating a functional connection between FGF signaling and CLDN1 expression in GIST.

## 3. Discussion

Over the last two decades, increasing evidence suggests that claudins play a crucial regulatory role in oncogenesis, tumor progression, and the response to conventional therapies. Indeed, the expression of proteins regulating tight junctions between epithelial cells decreases in parallel with cancer progression and is associated with higher tumor staging, poorer tumor histological grading, and unfavorable outcomes [45]. On the one hand, claudins inhibit cancer cell proliferation, invasion, and migration [46,47,48] and regulate stemness in cancer cells [49], thereby regulating the chemosensitivity of cancer. On the other hand, claudins exhibit opposite activities in cancer cells, enhancing their motility, proliferation, and invasion [50,51], as well as the self-renewal and activities of cancer stem-like cells [52,53]. Consequently, they are shown to be involved in tumor progression and metastasis in in vivo models [54].

Claudins, in particular, claudin-1 (CLDN1), has been frequently found to be overexpressed in primary cancers and metastasis, as well as in cancer cell lines [55,56], and the increased expression of CLDN1 was evidenced for a broad spectrum of human malignancies and cancer cell lines, including laryngeal squamous cell carcinomas (LSCCs) [57], colorectal cancer (CRC) [58], and breast and thyroid cancer [59,60,61]. In some cases, including breast cancer and CRC, the changes in expression of CLDN1 in tumors were shown to be associated with aggressive forms of diseases, larger tumor size, vascular invasion, higher pathological tumor stage, high metastatic lymph node ratio, and worse overall survival (OS) and progression-free survival (PFS) [61,62]. Notably, the increase of CLDN1 expression was detected following neoadjuvant chemotherapy [63] and associated with resistance to certain chemotherapeutic agents, including doxorubicin and cisplatin [30]. Thus, the increased expression of CLDNs in primary tumors makes them promising targets for cancer therapy, which is supported by the efficacy of specific monoclonal antibodies (e.g., Zolbetuximab, IMAB362, and claudiximab [64,65,66]), as well as small-molecule inhibitors [67].

The regulatory role of claudins in tumor resistance to the chemotherapeutic agents is well-documented for a broad spectrum of human malignancies, including pancreatic duct adenocarcinoma [42], lung cancer [29], and CRC [31]. The molecular mechanisms underlying CLDN1-mediated cancer chemoresistance are diverse and include its interaction with EPHA2 kinase, which promotes stemness [31], the upregulation of Unc-51-like autophagy activating kinase 1 (ULK1) phosphorylation [29], and significant alterations in metabolic pathways [42]. CLDN3, 4, and 6 were also shown as potent regulators of chemoresistance in various malignancies, including ovarian, lung, and breast cancer [32,33,34,35,36,37]. This might be due to the CLDNs’ interactions with both α- and β-tubulins, thereby affecting the structure and dynamic state of the microtubules [35], promoting cancer cell stemness [32,33,34], or regulating the Cu transporter CTR1 [36].

Despite the high impact of CLDNs in tumor progression and sensitivity to chemotherapies being well-documented and discussed in detail in several fundamental reviews [38,39,40], the effect of claudins in GIST pathogenesis, progression, and resistance to targeted therapies remains unknown.

We show here for the first time the increased expression of CLDN1 in IM-resistant GIST T-1R cells (Figure 1). This contributes to GIST resistance to IM both in vitro and in vivo. Indeed, increased expression of apoptotic markers and decreased cellular proliferation were observed in IM-resistant GIST T-1R cells treated with IM in combination with the CLDN1 inhibitor PDS-0330 or after *CLDN1* knockdown (Figure 2 and Figure 3). The high potency of the dual inhibition of KIT and CLDN1 signaling was also demonstrated in vivo. Indeed, the volumes of GIST xenografts dramatically decreased after the combined therapy of IM and PDS-0330 was used (Figure 9A,B). Notably, the histological examination of IM-resistant GIST revealed the high potency of PDS-0330 used alone, as evidenced by the increased size of the necrotic areas observed at the final time-point of the experiment (Figure 9C). This correlated with the decreased sizes and volumes of IM-resistant xenografts treated with PDS-0330 and was consistent with the effect of this CLDN1 inhibitor on CRC xenografts [67].

Given that the activation of the FGFR pathway was previously shown to be a potent mechanism of secondary resistance in GIST T-1R cells to IM [12], we examined the potential interplay between this pathway and CLDN1. The interaction between CLDN1 and FGFR2 in IM-resistant GIST was confirmed by immunofluorescence staining and co-immunoprecipitation data (Figure 6A and Figure 6B, respectively), thereby illustrating tight co-localization and interaction between CLDN1 and FGFR2. Moreover, we observed that FGF-2 ligand increased expression of CLDN1 in IM-naive GIST cells in a time-dependent manner (Figure 5A), whereas pan-FGFR inhibitors (e.g., AZD 4547 and TAS-120) decreased CLDN1 expression in IM-resistant GIST T-1R cells (Figure 5B). Strikingly, *CLDN1* knockdown significantly decreased the expression of FGFR2, but not FGFR1, 3, and 4 (Figure 5C). This might be due to the ability of CLDN1 to regulate FGFR2 stability by preventing its ubiquitination, endocytosis, and lysosomal degradation. Indeed, some surface proteins, including EMT-related proteins (e.g., N-cadherin), have been shown to be potent regulators of FGFR expression by preventing their degradation through the lysosomal pathway [68]. The CLDN1-mediated regulation of FGFR2 stability in GIST cells will be a subject for further studies.

Notably, the pattern above was specific to GIST cells lacking secondary *KIT* mutations (e.g., GIST T-1R cells), whereas we did not observe significant benefits of CLDN1 inhibition in GIST 430 cells. This was evidenced by the lack of synergy between IM and PDS-0330 (Figure 4) and by the induction of apoptosis in GIST 430 cells after the dual inhibition of KIT- and CLDN1-signaling (Figure 3). Besides the differences in *KIT* mutational status between these IM-resistant GIST cell lines, both of them exhibited similar levels of phospho-KIT/c-KIT and FGFR2, as well (Supplementary Figure S6). Based on our data, which illustrate a tight cross-talk between FGFR2 and CLDN1 (Figure 5, Figure 6 and Figure 8), CLDN1 may serve as a potent regulator of FGFR signaling in GIST, thereby maintaining resistance to IM and promoting disease progression. In agreement with this, increased expression of CLDN1 was observed in most primary GIST specimens from the high-risk group, as shown in Figure 12A,B, indicating that CLDN1 promotes an aggressive phenotype in GIST.

Collectively, we also showed here, for the first time, the impact of CLDN1 in GIST resistance to IM. These mechanisms underlie the FGFR-signaling pathway. Given that the activation of the FGFR-signaling pathway was previously shown by our group as a potent mechanism of secondary resistance to IM in GIST [12] and taking into account our present data illustrating a tight cross-talk between CLDN1 and FGFR-signaling (Figure 8), we conclude that CLDN1 regulates FGFR expression and signaling in IM-resistant GIST. In contrast, the knockdown of *CLDN1* or its inhibition effectively reverses GIST’s sensitivity to IM by interrupting the FGFR-mediated survival cascade (Figure 13).

This was also supported by transcriptome profiling data, which illustrated the significant impairment of AKT- and MAPK-signaling pathways in CLDN1-inhibited GIST (Figure 11C,D). Additionally, significant changes in metabolic pathways in both *CLDN1*-knockdowned or PDS-0330-treated GIST cells (Figure 11A,B) are in concordance with recent findings [42]. Lastly, transcriptome profiling of IM-resistant GIST T-1R cells with inhibited or knocked-down *CLDN1* (Figure 10B) illustrated the other potential targets that may be involved in GIST resistance to targeted therapies. This might include *AZIN1* (antizyme inhibitor 1), *SFRP1* (soluble frizzled related protein 1), or *CHST1* (carbohydrate sulfotransferase 1) (Figure 10B) that were previously shown to be involved in cancer resistance to certain chemotherapeutic agents (e.g., paclitaxel, doxorubicin and cisplatin) [69,70,71,72] and targeted therapies [73,74,75,76] as well. The molecular mechanisms of chemoresistance of cancer cells are complex and include the neutralization of ornithine decarboxylase antizymes [72], the regulation of cholesterol synthesis [73], the upregulation of P-glycoprotein and Bcl-2 [77], the activation of DNA damage repair [78], and c-Jun signaling, AXL transcription, and activation of the Erk-mediated pathway, as well. Further studies will help assess the contribution of the aforementioned factors in GIST resistance to IM.

Overall, our data illustrate the high potency of CLDN1 in maintaining the malignant phenotype of GIST and regulating their sensitivity to IM-based therapy. This was due to the physical interaction and functional partnership between CLDN1 and the FGFR2-mediated survival pathway observed in IM-resistant GIST. Therefore, a combination of IM and PDS-0330, a specific CLDN1 inhibitor, acted synergistically to induce apoptotic cell death and decrease the proliferative rate of GIST in vitro, and it effectively blocked tumor growth in the GIST xenograft model. Notably, this was specific to GIST T-1R cells that overexpressed CLDN1 and lacked secondary *KIT* mutations. In contrast, GIST 430 cells, which harbored secondary *KIT* mutations and lacked CLDN1 expression, remained non-sensitive to the aforementioned dual inhibition. This, in turn, might open novel therapeutic opportunities in the management of a specific subset of GIST by targeting CLDN1.

## 4. Materials and Methods

### 4.1. Chemical Compounds

Imatinib mesylate (IM) and PDS-0330 were obtained from Selleck Chem (Houston, TX, USA).

### 4.2. Antibodies

Primary antibodies used for immunoblotting and immunofluorescence were as follows: phospho-KIT Y719 (#3391S, lot: 12), AKT (#4691T, lot:28), cleaved form of caspase-3 (#9664S, lot:22), cleaved form of PARP (#5625S, lot:13), claudin-1 (#13255S, lot:5), calnexin (# 2679T, lot:6), Brg1 (3508T, lot:2), E-cadherin (#3195T, lot:15), N-cadherin (#13116T, lot:6), Vimentin (#5741T, lot:8), Beta-catenin (#8480T, lot:9), FGFR1 (#9740S, lot:4), FGFR2 (#23328S, lot:3), FGFR3 (#4574S, lot:2) (Cell Signaling, Danvers, MA, USA), KIT (#A4502, lot:11164713, Dako, Carpinteria, CA, USA), beta-actin (A00730-200, lot:20A002063, GenScript, Piscataway, NJ, USA), FGFR4 (#SAB1300019-100UG, lot:129K0498, Sigma-Aldrich, St-Louis, MO, USA), and claudin-1 (sc-166338, lot:F2624, Santa Cruz Biotechnology, Santa Cruz, CA, USA). For co-immunoprecipitation (co-IP) assays, the aforementioned anti-FGFR1 and 2 monoclonal Abs were utilized. Additionally, cell lysates were precipitated for CLDN1 by using the claudin-1 mAbs (sc-166338, lot:F2624, Santa Cruz Biotechnology, Santa Cruz, CA, USA) or FGFR2-precipitating Abs (ab281925, clone number:EPR24679-79, Abcam, Cambridge, UK). HRP-conjugated secondary antibodies for Western blotting were purchased from Santa Cruz. Neutralizing monoclonal antibodies to FGF-2 (Anti-FGF2 mAbs, #05-117, lot:3282554) were obtained from Merck KGaA (Darmstadt, Germany).

### 4.3. Cell Lines and Culture Conditions

The GIST T-1 cell line was established from a metastatic pleural tumor from stomach GIST and contains a heterozygous 57-base pair deletion (V570-Y578) in *KIT* exon 11 [79]. IM-resistant GIST T-1R subline was established in our laboratory after continuous induction from 0.4 nM to 1000 nM IM in a stepwise increasing concentration manner [12]. The IM-resistant GIST 430 cell line was derived from a human GIST that developed clinical resistance to IM-based therapy. This cell line carries a heterozygous primary *KIT* exon 11 deletion (V560_L576del) and a secondary *KIT* exon 13 point mutation (V654A) [79]. GIST cell lines were grown in a humidified atmosphere of 5% CO_2_ at 37 °C (LamSystems, Myass, Russia).

### 4.4. Cellular Survival MTS-Based Assay

To examine the cytotoxicity of RTKis, GIST cells were seeded in 96-well flat-bottomed plates (Corning Inc., Corning, NY, USA) and allowed to attach and grow for 24 h. The cells were then cultured for 24–48 h with the indicated concentrations of the RTKis or DMSO (control). Finally, MTS reagent (Promega, Madison, WI, USA) was added to the culture medium for at least one hour to assess the live cell numbers. Cellular viability was assayed at 492 nm using a MultiScan FC plate reader (Thermo Fisher Scientific, Waltham, MA, USA). The resulting IC_50_ values were defined as the concentration of the compound required to inhibit cellular growth by 50% within 24–48 h. The data were normalized to the DMSO-treated control group. IC_50_ values were determined using the IC_50_ Tool Kit ( https://ic50.org/, accessed on 23 July 2024).

The experiments were performed at least in triplicate, and the potential additive, antagonistic, and synergistic effects of PDS-0330 on IM were calculated by using the R package of the computational tool Synergy Finder (https://bioconductor.org/packages/release/bioc/html/synergyfinder.html, accessed on 1 August 2024). For this purpose, the following four types of models were used: the highest single agent (HSA) model [80], the Loewe additivity model [81], the Bliss independence model [82], and the zero interaction potency (ZIP) model [83]. A value of synergy score (SC) of PDS-0330 and IM combinations < –10 was considered an antagonistic effect, whereas SCs between −10 and 10 and SCs > 10 were considered an additive and synergistic effect, respectively [83].

### 4.5. Real-Time Monitoring of Cell Proliferation

GIST cells (0.5 × 10^5^ /mL) were seeded into the wells of an E-Plate L8 PET cassette (ACEA Biosciences, San Diego, CA, USA). The cassettes were installed in the iCELLigence cell growth kinetics system (ACEA Biosciences, San Diego, CA, USA). Cells were allowed to attach and grow for the following 24 h. Subsequently, IM 1 µM PDS-0330 5 µM, alone or in combination, were introduced into cell culture. DMSO-treated cells served as the control. Additionally, the proliferation of GIST T1-R cells with *CLDN1* knockdown (alone and in the presence of 1 µM IM) was studied. Cell proliferation index values were recorded every hour throughout the experiment. The experiments were performed at least in triplicate. RTCA Software version 1.0 (ACEA Biosciences, Inc., San Diego, CA, USA) was used to analyze the data.

### 4.6. Crystal Violet Staining

For staining, a crystal violet fixing solution of the following composition was used (per 50 mL dH2O): 25 mg crystal violet, 3.1 mL of 16% formaldehyde solution, 5 mL of PBS (10×), and 0.5 mL of 100% methanol. The medium was removed from the culture dishes, and a crystal violet fixative solution was added (3 mL for dish p60 and 6 mL for dish p100). It was incubated for 20 min at room temperature in the dark. Next, the dishes were washed from the fixing solution in a container with cool water for 2–3 min and left to dry on filter paper for 2–3 h at room temperature. Afterward, the dishes were photographed. To quantify crystal violet staining, 1% SDS solution was added to the dishes (1.5 mL solution for dish p60, 3 mL for dish p100) and incubated for 1 h at room temperature on a shaker. Next, 100 μL of the solution from the dishes was added to the wells of a 96-well plate, and the optical density was measured at 540 nm on a MultiScan FC plate reader (Thermo Fisher Scientific, Waltham, MA, USA).

### 4.7. Subcellular Fractionation

Cellular fractionations were performed with commercial extraction buffers in the Subcellular Proteome Extraction Kit (S-PEK) (Calbiochem, Merck, Darmstadt, Germany). The GIST cell suspension (3–5 × 10^6^/mL) was washed twice with 2 mL of wash buffer and centrifuged at 300× *g* at 4 °C for 10 min. In the first step, with Extraction Buffer I and an inhibitor cocktail, the cytosolic proteins were incubated for 10 min at 4 °C, followed by centrifugation for 10 min at 1000× *g*. Membranes and membrane organelles were solubilized with Extraction Buffer II and an inhibitor cocktail, incubated for 30 min at 4 °C, and then centrifuged for 10 min at 6000× *g*. Next, nucleic proteins were enriched with Extraction Buffer III, inhibitor cocktail, and benzonase, incubated for 10 min at 4 °C, and centrifuged for 10 min at 10,000× *g*. The experiments were performed at least in triplicate, and assessment of protein expression in the fractions mentioned above was performed by Western blotting, as indicated below.

### 4.8. siRNA-Mediated Knockdown of CLDN1 and FGFR2

The knockdown of *CLDN1* and *FGFR2* was performed using a SMARTpool (Dharmacon, Lafayette, CO, USA). A non-targeting siRNA (Dharmacon, Lafayette, CO, USA) was used for a negative control. Briefly, 10 μL of Lipofectamine 2000 (Thermo Fisher Scientific, Waltham, MA, USA) and 10 μL of siRNA (20 μM) or negative control were mixed with Opti-MEM (Gibco, New York, NY, USA) to a final volume of 400 μL per well at room temperature for 30 min according to the manufacturer’s protocol. Next, a transfection mix was introduced into 70% confluent GIST T-1R culture for 10 h. After the mix was removed, the cells were cultured in a complete culture medium for 48 h before use in functional assays, Western blotting, or RNA analysis.

### 4.9. Western Blotting

For Western blotting analysis, whole-cell extracts (WCE) were prepared by scraping the cells growing as monolayers into RIPA buffer (25 mM Tris-HCl pH 7.6, 150 mM NaCl, 5 mM EDTA, 1% NP-40, 1% sodium deoxycholate, 0.1% SDS) and were supplemented with protease and phosphatase inhibitors. The cellular lysates were incubated for one hour at 4 °C and then clarified by centrifugation for 30 min at 13,000 rpm at 4 °C. Protein concentrations were measured using the Bradford assay. The samples containing 30 μg of protein were resolved on 4 to 12% Bis-Tris or 3 to 8% Tris-acetate NuPAGE gels (Invitrogen, Carlsbad, CA, USA), transferred to a nitrocellulose membrane (Bio-Rad, Hercules, CA, USA), probed with a specific antibody, and visualized by enhanced chemiluminescence (Western Lightning Plus-ECL reagent, Perkin Elmer, Waltham, MA, USA). Densitometry analysis of Western blotting images was performed using the NIH Image J software (version 1.49) (Bethesda, MD, USA).

### 4.10. Co-Immunoprecipitation (Co-IP)

For Co-IP, whole cell lysates (WCL) were prepared by scraping the cells into PBS 1X and centrifuged for 3 min at 13,000 rpm at +4 °C on ice (10 min) using TEB buffer (50 mM Tris-HCl pH 7.5, 150 mM NaCl, 1% NP-40, 10% glycerol) supplemented with a protease inhibitor cocktail solution and PMSF (Sigma-Aldrich, St-Louis, MO, USA). The samples were then centrifuged for 10 min at 13,000 rpm at +4 °C to obtain the supernatants (whole-cell lysates). Claudin-1 precipitating antibodies (sc-166338, lot:F2624, Santa Cruz Biotechnology, Santa Cruz, CA, USA), at a concentration of 2 μg, or FGFR2 precipitating Abs (ab281925, clone number: EPR24679-79, Abcam, Cambridge, UK) were added to WCL and incubated overnight at +4 °C on a shake and further loaded by 30 μL of protein A and G Sepharose beads (Santa Cruz Biotechnology, Santa Cruz, CA, USA) and incubated on a shaker for 1 h at +4 °C. After washing with TEB buffer, a mixture of LDS sample buffer (Invitrogen, Carlsbad, CA, USA) and 2-mercaptoethanol (Sigma-Aldrich, St-Louis, MO, USA) was added to the samples. The samples were further subjected to vertical electrophoresis and blotting, as shown before. The experiments were performed at least in triplicates.

### 4.11. Immunofluorescence Staining

GIST cells (0.5 × 10^5^/mL) were seeded into the 6-well flat-bottom plates (Corning Inc., Corning, NY, USA) containing Poly-L-lysine-coated coverslips (Sigma-Aldrich, St. Louis, MO, USA). Over the next 48 h, the cells were allowed to attach and grow. Next, the cells were incubated for 48 h in the presence of DMSO (control), IM at 1 µM, PDS-0330 at 5 µM, and their combination. The cells were further fixed with a 4% paraformaldehyde solution (in PBS) for 15 min at room temperature. After washing with PBS, the glass coverslips were incubated in a blocking solution (1×PBS, 5% goat serum, 0.3% Triton X-100) for one hour at room temperature. After this, the cells were incubated with primary antibodies to the CLDN1 (sc-166338, lot:F2624, Santa Cruz Biotechnology, Santa Cruz, CA, USA) and FGFR2 (#23328S, lot:3, Cell Signaling, Danvers, MA, USA) and were dissolved in a ratio of 1:200 in antibody dilution buffer (1× PBS, 1% BSA, 0.3% Triton X-100) overnight at 4 °C. The next day, the cells were washed with PBS (three times for 5 min each in the dark), incubated with Alexa Fluor 488 (A11008, lot:1885240)- or Texas Red (T6390, lot:2480080)-conjugated secondary antibodies (Invitrogen, Carlsbad, CA, USA), and dissolved in a ratio of 1:500 in antibody dilution buffer for one hour at room temperature in the dark. Next, the cells were washed three times with PBS (5 min each in the dark) and then stained with a DAPI solution (Sigma-Aldrich, St. Louis, MO, USA) for 30 s. After washing with PBS, the coverslips were placed on the glass slides, and cells were visualized using an Olympus BX63 fluorescence microscope (Tokyo, Japan). Images were acquired using the Spot Advanced Imaging System. The images were examined in at least 5 fields of view, and ~70–80 cells were captured for each particular experimental setting. The experiments were performed at least in triplicate.

### 4.12. RNA Extraction and Real-Time Quantitative PCR

Total RNA was extracted from GIST T-1 and GIST T-1R cells and converted into complementary DNA (cDNA) as previously described [12]. One µL template cDNA was used in a real-time qPCR reaction with 5× qPCRmix-HS SYBR (PB025, Evrogen, Moscow, Russia) and 10 mM each forward and reverse primers for experimental or control genes (Supplementary Table S2). According to the manufacturer’s protocol, real-time qPCR was performed using the CFX96 Real-Time Detection System (Bio-Rad, Hercules, CA, USA). The relative levels of each mRNA were normalized to those of *ACTB*. Quantitative data were generated based on the number of cycles required for the fluorescence generated by amplification to reach a specific threshold of detection (the Ct value). The Western blot images of CLDN1 and actin expression were processed using the Image J program (version 1.49). Then, the obtained densitometry values were normalized to actin. Afterwards, the CLDN1 expression in each GIST sample was statistically analyzed.

### 4.13. RNA-Seq Library Preparation, Sequencing, and Bioinformatics Pipeline

Cells were collected by centrifugation, washed with ice-cold PBS, and immediately deep-frozen in liquid nitrogen. Cellular pellets were used for total RNA isolation using the RNeasy Mini Kit (Qiagen, Hilden, Germany) according to the manufacturer’s instructions.

The quality of total RNA was evaluated using the Bioanalyzer 2100 (Agilent, Santa Clara, CA, USA). The quantity and purity of RNA were estimated on a NanoPhotometer (Implen), and 800 ng of RIN ≥ 7 of total RNA was used for library construction using NEBNext^®^ Poly(A) mRNA Magnetic Isolation Module and NEBNext^®^ Ultra II™ Directional RNA Library Prep Kit for Illumina (New England Biolabs, Ipswich, MA, USA), according to the manufacturer’s instruction. The quality of the libraries was verified using the Bioanalyzer 2100 (Agilent, Santa Clara, CA, USA), and the yield was validated by quantitative PCR (qPCR). Libraries were then sequenced on the NovaSeq 6000 (Illumina, San Diego, CA, USA) with paired-end 61 bp reads.

The quality of the sequenced reads was assessed using FastQC v0.11.5. Unwanted adapter and rRNA matching reads were filtered out using Trimmomatic at a threshold of 0.38. Trimmed reads were aligned to human genome assembly hg38 (UCSC primary chromosomes) with Hisat2 v2.1.0 and counted with HTSeq v2.0.1 against Gencode v43 basic annotation. Further analyses, including FPKM, TMM normalization, differential expression, and functional enrichment, were conducted in the R environment (packages edgeR v3.42.2 and cluster Profiler v4.8.1). We consider the significance threshold for differentially expressed genes to be FDR < 0.05 and a fold change of >2, unless specific parameters are specified. Raw and processed transcriptome data were accessible through GEO NCBI under the ID GSE247170.

### 4.14. GIST Xenograft Studies

Subcutaneous human tumor xenografts were generated via s.c. inoculation in the flank areas of 5- to 8-week-old female nu/nu mice with 100 μL of 5 × 10^6^ IM-naive (GIST T-1) and IM-resistant (GIST-T-1R) cells/mL suspensions in Dulbecco’s phosphate-buffered saline. The animal experimental protocols were approved by the Committee for Ethics of Animal Experimentation, and the experiments were conducted under the Guidelines for Animal Experiments in N.N. Blokhin National Medical Research Center of Oncology. All s.c. tumors were allowed to reach ~400 mm^3^ volumes (day 14) before randomization of mice into four groups (n = 4). They administered i.p. 100 μL of vehicle (negative control), PDS-0330 (5 mg/kg), IM (50 mg/kg), or a combination of PDS-0330 and IM three times a week for 2 weeks. The changes in tumor sizes were calculated as a percentage of the baseline. The tumor volume in each group was assessed by calipers, calculated as length × width × width × 0.5, and tumor growth curves were drawn accordingly. Results were expressed as the mean tumor volume compared to control (vehicle-treated) animals. After the mice were sacrificed, the tumors were isolated, photographed, weighed, subjected to histopathologic examination, and also prepared to assess caspase 3/7 activity.

Formalin-fixed, paraffin-embedded (FFPE) tissues were sectioned at 4 μM for hematoxylin and eosin (H&E) staining. The images of the stained samples were captured using an Olympus BX63 microscope (Olympus, Tokyo, Japan).

Caspase-3/7 activity in xenografts was measured by using the caspase-Glo 3/7 Assay (Promega, Madison, WI, USA) kit. Protein concentrations in the samples were determined using the Bradford assay. Caspase-Glo 3/7 Reagent was added to lysates for 2 h at a 1:1 ratio at room temperature, and the luminescence intensity was assessed at 570 nm by using a microplate reader.

### 4.15. Statistics

All the experiments were repeated a minimum of 3 times. The Shapiro–Wilk test was used to assess normality. Normally distributed data were presented as mean ± standard deviation for each group. Differences were considered significant at *p* < 0.05 (*) using an unpaired Student’s *t*-test.

A two-tailed Fisher’s exact test was used to examine the significance of the relationship between two variables in a contingency table (in particular, between CLDN1 expression and tumor location/gender/prognostic group). Differences were considered significant at *p* < 0.05 (*).

## Figures and Tables

**Table 1:** Synergy scores (SC) between IM and PDS-0330 in IM-resistant GIST T-1R and GIST 430 cells.

Cell Line	ZIP	Bliss	Loewe	HSA
GIST T-1R	14.22	14.20	19.38	19.03
GIST 430				2.02

Results were obtained by calculating the synergy scores using four different computational tools (ZIP model, Bliss independence model, Loewe additivity model, and HSA model) via the R package Synergy Finder. A value of SC of PDS-0330 and IM combinations < −10 was considered an antagonistic effect, whereas SCs between −10 and 10 and SCs > 10 were considered an additive and synergistic effect, respectively.

**Table 2:** Descriptive and analytical statistical data of two comparison groups (low risk/intermediate and high risk).

	Low Risk	Intermediate/High Risk	*p*
Mean	3.60	6.29	0.011
Standard deviation	2.03	2.65	
Number of patients, n	12	12	

## Data Availability

The original contributions presented in this study are included in the article/supplementary material. Further inquiries can be directed to the corresponding author(s).

## Supplementary Materials

The following supporting information can be downloaded at: https://www.mdpi.com/article/10.3390/ijms26178138/s1. Refs. [84,85,86,87] are cited in Supplementary Materials.
