# Supplementary material for: Claudin-1 Contributes to Gastrointestinal Stromal Tumors (GIST) Resistance to Imatinib Mesylate (IM) via Regulation of FGFR-Signaling
Source: Int J Mol Sci. 2025 Aug 22;26(17):8138. doi: 10.3390/ijms26178138 (PMC12428531; doi:10.3390/ijms26178138)
Supplement: Supplementary file 1 [file ijms-26-08138-s001.zip › ijms-3767321-supplementary.pdf]

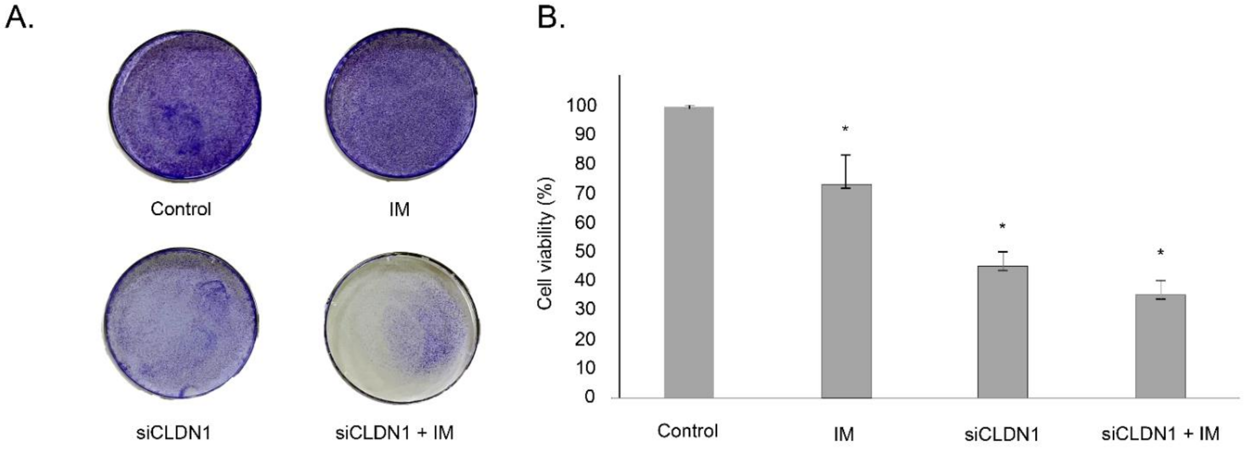

Supplementary Figure S1. (A) Representative images of crystal violet staining of GIST-T1R cells knockdown of CLDN1 and treated with IM alone or in combination. After the medium was removed from the culture dishes, crystal violet fixative solution was added for 20 minutes. Next, the dishes were washed from the fixing solution and left to dry on filter paper. Afterward, the dishes were photographed. **(B)** Quantification of crystal violet staining of GIST cells. To quantify crystal violet staining, 1% SDS solution was added to the dishes and incubated for 1 hour at room temperature on a shaker. Next, 100 µl of the solution from the dishes was added to the wells of a 96-well plate, and the optical density was measured at 540 nm on a MultiScan FC plate reader (Thermo Fisher Scientific, Waltham, MA, USA). Significant differences in compare with control * $p \leq 0.001$ from $n \geq 3$ using unpaired Student's t-test.

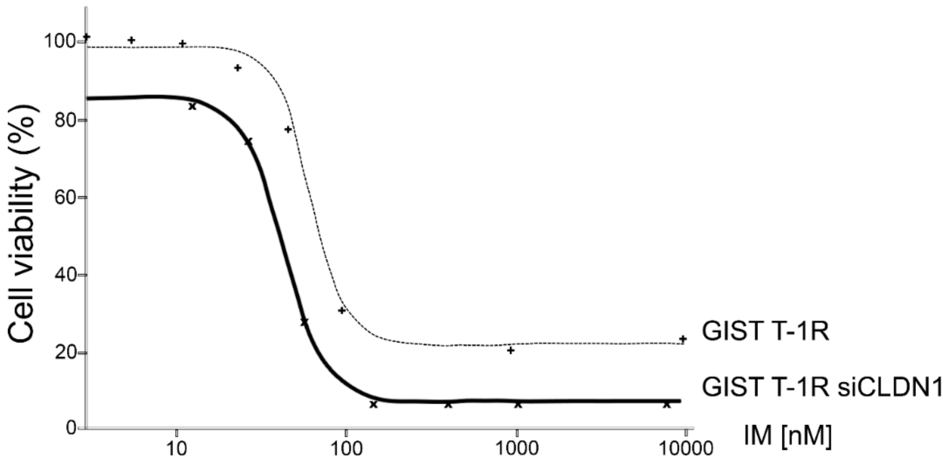

Supplementary Figure S2. MTS-based viability assay in mock-transfected IM-resistant GIST-T1R cells and transfected with siRNA CLDN1 for 48h. The cells were further treated with indicated concentrations of IM and viability was assessed after 48 h of treatment. The data was normalized to mock-transfected controls. Values are the means ± standard deviation (n = 4).

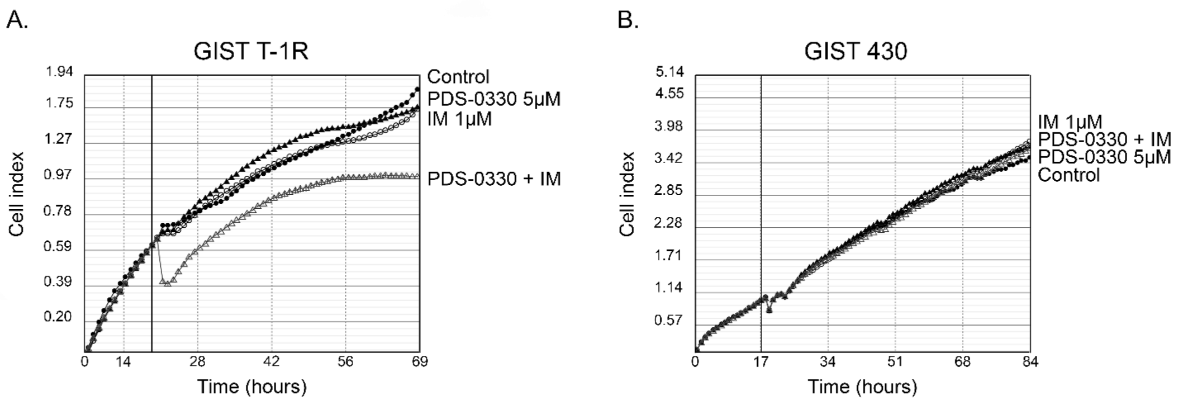

Supplementary Figure S3. Changes in growth kinetics of GIST-T1R cells **(A)** and GIST 430 cells **(B)** treated with DMSO (control), IM, PDS-0330 alone and in combination.

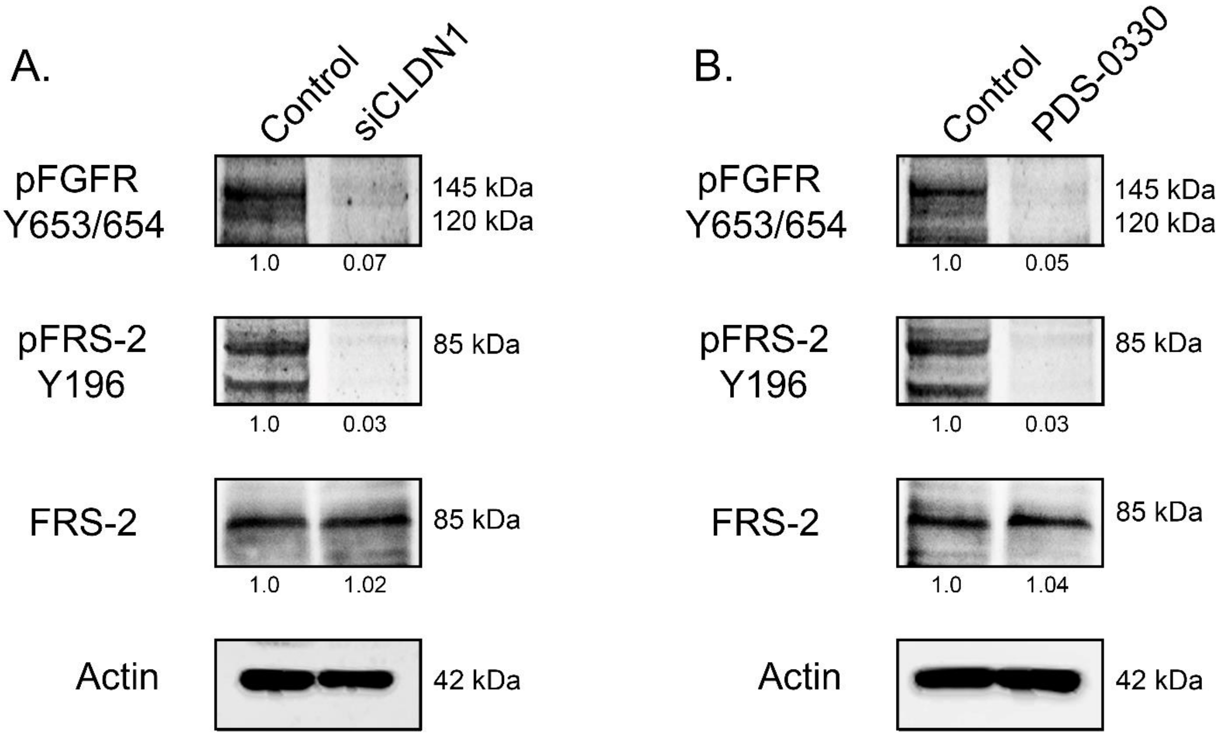

Supplementary Figure S4. CLDN1 inhibition abrogates activation of FGFR-pathway in IM-resistant GIST. GIST T-1R cells were transfected with siCLDN1 **(A)** or treated by CLDN1 inhibitor PDS-0330 (5 μM) **(B)**. Control cells were transfected with scrambled siRNA or treated with solvent (e.g., DMSO). The lysates were subjected to WB analysis to examine expression of the total and phosphorylated forms of FGFR and FRS-2. Actin staining was used to demonstrate comparable amounts of protein loaded into each sample.

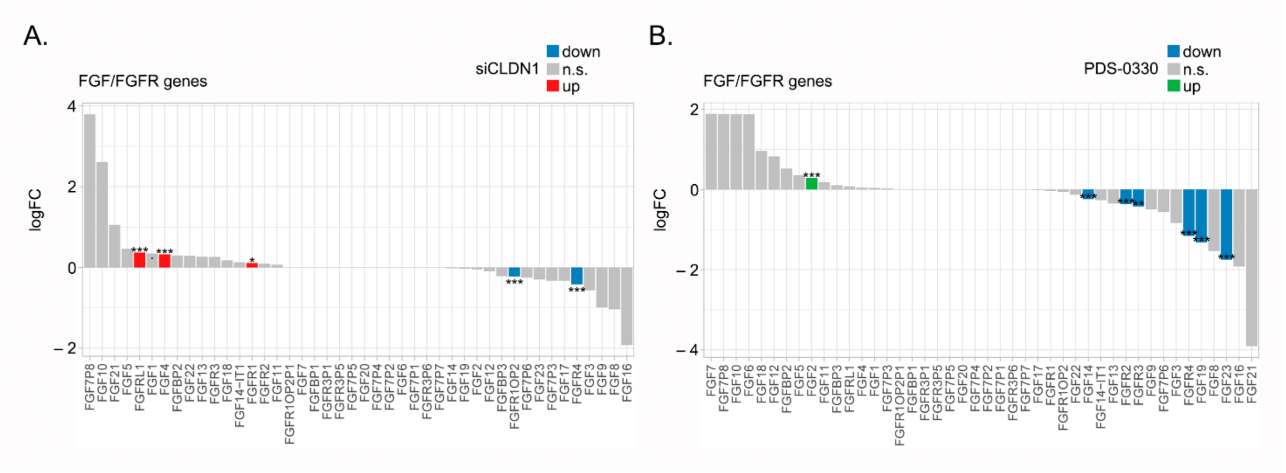

Supplementary Figure S5. Gene expression changes in FGF signaling pathways in GIST T-1R cells transfected with siRNA CLDN1 **(A)** and GIST T-1R cells treated with PDS-0330 **(B)**. *** FDR < 0.001, ** FDR 0.001–0.01, * FDR 0.01–0.05, not significant FDR>0.05.

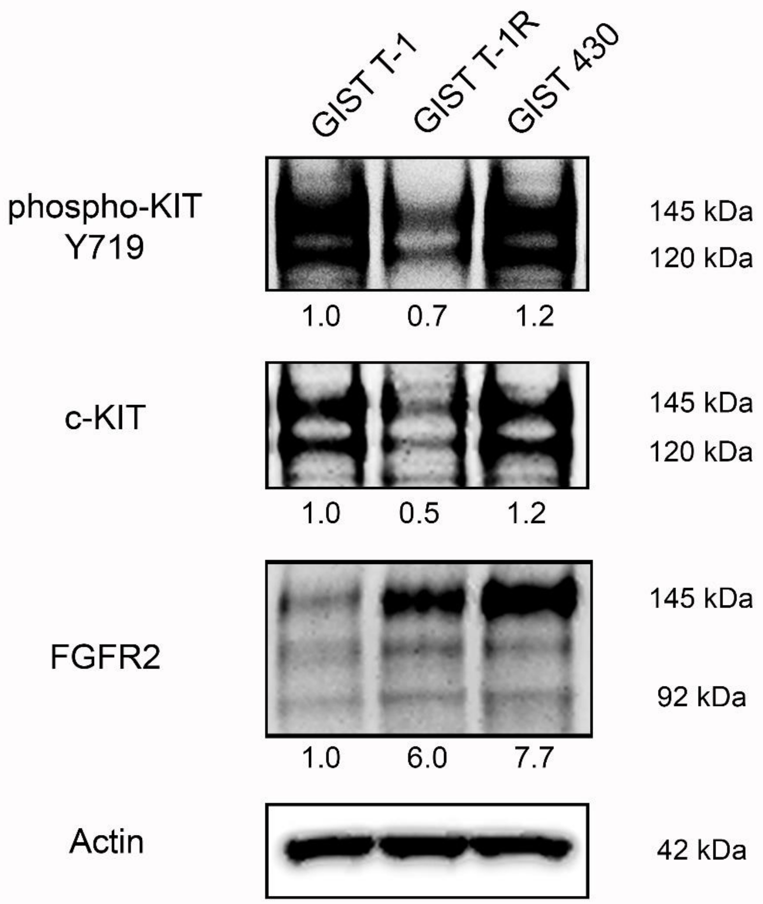

Supplementary Figure S6. Expression of phospho-KIT Y719, c-KIT and FGFR2 in IM-naive (GIST T-1) and IM-resistant (GIST T-1R, GIST 430) cells. Actin staining was used to show the comparable amounts of protein loaded into each sample.

Supplementary Table S1. The characteristics of GIST patients enrolled in present study.

№	Age	Gender	Location	KIT expression	Prognostic group *
1	69	f	stomach	+	2
2	68	f	stomach	+	2
3	50	f	stomach	+	2
4	71	f	stomach	+	2
5	59	f	small bowel	+	2
6	68	f	lesser omentum	+	2
7	72	f	stomach	+	6
8	73	f	stomach, mts in the liver	+	6
9	63	f	stomach, mts in the liver	+	6
10	71	m	stomach	+	6
11	46	f	stomach	+	6
12	70	f	stomach	+	6
13	58	f	stomach	+	2
14	47	f	stomach	+	2
15	63	f	stomach	+	1
16	41	m	stomach	+	2
17	76	m	stomach	−	2

18	61	f	small bowel	+	2
19	78	m	stomach	+	3
20	64	f	stomach	+	5
21	33	f	stomach	+	5
22	68	f	stomach	−	6
23	41	m	stomach	+	6
24	67	m	small bowel	+	6

** Low risk − 1-2 prognostic group, intermediate risk − 3-4 prognostic group, high risk − 5-6 prognostic group.*

Supplementary Table S2. Primers used for real time qPCR

Gene (protein)	Forward sequence	Reverse sequence	Source
CDH2 (N-cadherin)	GACAATGCCCCTCAAGTGTT	CCATTAAGCCGAGTGATGGT	[84]
CDH1 (E-cadherin)	GCCTCCTGAAAAGAGAGTGGAAG	TGGCAGTGTCTCTCCAAATCCG	[85]
VIM (Vimentin)	AGGCAAAGCAGGAGTCCACTGA	ATCTGGCGTTCCAGGGACTCAT	
SNAIL1 (Snail Family Transcriptional Repressor 1)	TGCCCTCAAGATGCACATCCGA	GGGACAGGAGAAGGGCTTCTC	
CLDN1 (Claudin 1)	TGGTATAGCCCACCAGAAAGGAC	CAATCCCGCTATTGTGGTTTCCG	[86]
ACTB (Actin Beta)	AGCACAGAGCCTCGCCTT	CATCATCCATGGTGAGCTGG	[87]
